# Nutrition Support Interventions for Children and Young People Treated for Osteosarcoma: A Scoping Review

**DOI:** 10.1111/jhn.70172

**Published:** 2025-11-28

**Authors:** Laura Sealy, Maddie Smith, Sarah Barrett, Divyalakshmi Bhaskaran, Helen Rees, Raquel Revuelta Iniesta

**Affiliations:** ^1^ Department of Paediatric Nutrition and Dietetics Bristol Royal Hospital for Children Bristol UK; ^2^ Children's Health and Exercise Centre, Faculty of Public Health and Sports Sciences, Medical School University of Exeter Exeter UK; ^3^ Library and Knowledge Service University Hospitals Bristol & Weston NHS Foundation Trust Bristol UK; ^4^ Leeds Institute of Medical Research, St. James' Institute of Oncology, Bradford Teaching Hospitals NHS Foundation Trust University of Leeds Leeds UK; ^5^ Department of Paediatric Oncology Bristol Royal Hospital for Children Bristol UK

**Keywords:** gastrostomy, micronutrient, nasogastric, nutrition support, osteosarcoma, tube feeding, undernutrition

## Abstract

**Objective:**

To map evidence for nutrition interventions during osteosarcoma (OS) treatment for children and young people (CYP), identifying gaps to inform research for this predominantly adolescent group.

**Design:**

Scoping review following Joanna Briggs Institute methodology and PRISMA‐ScR guidelines.

**Data Sources:**

MEDLINE, EMBASE, CINAHL, EMCARE and CENTRAL were searched from inception to July 2025 using a three‐step search strategy (pilot, full, reference screening).

**Eligibility Criteria:**

Studies were included if they assessed nutrition interventions in patients with OS aged < 25 years undergoing treatment. Mixed cancer cohorts were eligible if > 50% had OS or OS data were reported separately. Exclusions: qualitative studies, patients > 25 years, survivors, animal or in vitro studies.

**Data Extraction and Synthesis:**

Four reviewers independently screened studies. Data on methodologies, nutrition interventions, outcomes and study design limitations were extracted and reported descriptively.

**Results:**

Of 7874 records retrieved, 15 studies (2001–2024) from 4 high‐ and 4 middle‐income countries met inclusion, involving 964 participants (median age 13.3 years), including 5 conference abstracts. Ten studies addressed comprehensive nutrition support (macro‐ and micronutrients), mostly using retrospective cohorts, while five focused on micronutrients. Common interventions included parenteral nutrition (5/15) and early gastrostomy feeding (5/15). Ten peer‐reviewed studies were fully characterised against the review objectives: six examined appetite stimulants, oral, enteral, and parenteral nutrition, or compared early versus reactive gastrostomy, reporting outcomes mainly via BMI *Z*‐scores; four reported gastrostomy complications. Two case reports described thiamine deficiency, and two RCTs assessed magnesium and vitamin D supplementation in relation to febrile neutropenia and survival. Key limitations included small cohorts, incomplete intervention reporting and unvalidated outcome measures.

**Conclusions:**

Research on nutrition interventions in CYP with OS is scarce, heterogeneous and methodologically weak. Large, prospective, multi‐centre studies are urgently needed to establish evidence‐based nutrition support strategies, valid outcome measures, and to examine the psychological impact of tube feeding for young people.

## Summary

- What is already known?
  ◦ Children and young people undergoing treatment for osteosarcoma experience intensive therapy that significantly impacts nutritional status, which in turn can influence treatment tolerance, immunity, quality of life and survival.
- What this study adds?
  ◦ Evidence on nutrition support during osteosarcoma treatment for children and young people is limited, fragmented and methodologically weak, but has increased in recent years.
  ◦ There is wide variation in clinical practice, particularly around early versus reactive gastrostomy placement, with unclear physiological and psychological benefits and risks.
  ◦ Whilst a range of nutrition support interventions have been studied, optimal nutrition support strategies for those undergoing treatment for osteosarcoma is unknown.
- How this study might affect research, practice or policy?
  ◦ It identifies priority research to establish evidence‐based clinical practice guidance: standardised, validated outcomes in prospective, multi‐centre and/or mixed methods studies.

## Graphical abstract

Whilst a range of nutrition support interventions for those undergoing osteosarcoma treatment has been studied and interest is growing, the evidence is fragmented, methodologically weak and indicates wide variation in clinical practice. We identify priority research needed to establish evidence‐based clinical practice guidance.

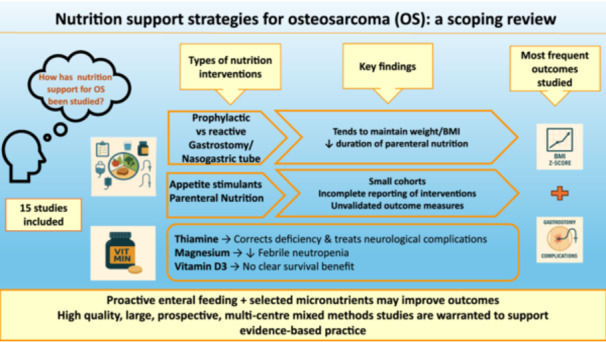

**Abbreviations**
aD3: active vitamin D3
BMI: body mass index
CNI: comprehensive nutrition intervention
CYP: children and young people
EN: enteral nutrition
FN: febrile neutropenia
GT: gastrostomy
JBI: Joanna Briggs Institute
MUAC: mid‐upper arm circumference
NG: nasogastric
OS: osteosarcoma
PN: parenteral nutrition
RIG: radiologically inserted gastrostomy

## 1 Introduction

Nutrition during cancer treatment is important [1]. High‐intensity treatment regimens for cancers, such as OS, are associated with undernutrition [2]; in turn, poor nutritional status at diagnosis and during treatment can increase treatment‐related toxicity, immunity, physical resilience, quality of life and survival [1, 3].

OS is the most common primary malignant bone tumour in children and young people (CYP) [4]. International registry data report 3–5 cases per million in males and 2–4 per million in females under 24, and a male:female ratio of 1.43:1. Typically arising in long bone metaphyses during the adolescent growth spurt, incidence peaks at ages 15–19 in males and 10–14 in females [5].

Current curative treatment generally includes neoadjuvant chemotherapy, surgical resection and adjuvant chemotherapy. In high‐income countries, this commonly comprises high‐dose methotrexate, doxorubicin and cisplatin [6]. Low‐income settings may omit high‐dose methotrexate due to resource constraints [7]. Multimodal therapy has improved survival from 10%–20% to ~60%; however, this has plateaued over the past 25–30 years [4, 5]. Five‐year survival rates are similar across European countries, Japan and United States (51%–75%) with some exceptions (Slovakia, Estonia and Denmark) [5]. Treatment remains classified as ‘very intensive’ (Level 3 of 4 on the ITR‐3 scale) [8].

Malnutrition in CYP results from inadequate or imbalanced nutrient intake, absorption, or utilisation, impairing growth, development and clinical outcomes [9]. It includes both undernutrition (e.g., wasting, stunting, underweight, micronutrient deficiencies) and overnutrition (overweight and obesity with or without micronutrient imbalance) [9]. CYP with OS can be particularly vulnerable to undernutrition due to the intensity of therapy, side effects (e.g., nausea, mucositis, anorexia) and the metabolic demands of disease and recovery [2].

In low‐ and middle‐income countries—home to ~90% of the global childhood cancer population—malnutrition contributes to treatment‐related toxicity, infections, longer length of hospital stay, therapy abandonment and mortality [10]. The dual burden of malnutrition and infectious diseases complicates OS diagnosis and reporting [10]. In high‐income nations, a low body mass index (BMI) in OS is associated with wound infections and slough [11], and could potentially impair participation in post‐operative rehabilitation, whilst a high BMI is associated with nephrotoxicity [3] and inferior prognosis [12].

For OS, nutrition support (defined as the administration of nutrients in place of, or in addition to, normal eating [13]) remains poorly characterised and inconsistently addressed, as for many paediatric cancers. This knowledge gap was highlighted by a Cochrane systematic review and meta‐analysis 10 years ago [13]. Childhood cancers are rare, resulting in small diagnostic cohorts. Many nutrition studies span multiple cancer types, despite differing pathophysiologies and treatment modalities, often lacking the statistical power to draw clear conclusions for individual diagnoses and treatments [13], such as OS [14]. This is despite recommendations to treat each cancer type distinctly in nutritional research [2]. Consequently, there is no consensus on optimal assessment of nutritional risk or delivery of tailored nutrition support during OS treatment. The developing and dynamic communication preferences and treatment decision‐related information needs of adolescents adds a layer of complexity to the choice of best nutritional supportive care strategies for this malignancy [15, 16].

Through this scoping review, we aim to map existing literature on the nature of nutrition support intervention studies for CYP with OS, to identify evidence gaps, and priorities for future research to support the development of clinical guidance to improve nutritional, clinical and quality‐of‐life outcomes for this vulnerable group.

Objectives/research questions:

1. What methodologies and designs have been used in studies on nutrition support interventions for osteosarcoma?
2. What are the characteristics of the nutrition support interventions?
3. What outcomes are assessed and how?
4. What are the limitations of the included study designs?

## 2 Methods

The aim comprises an indication for a scoping review methodology [17]. A preliminary search of MEDLINE, the Cochrane Database of Systematic Reviews and JBI Evidence Synthesis (23/01/2024) indicated no current reviews on the topic. There is insufficient evidence for a systematic review.

We followed JBI methodology for scoping reviews [18] and the PRISMA‐SCR extension [19]. Although not routinely undertaken within scoping reviews [19], we also appraised the methodological quality of included sources to complete the objective of identifying limitations and to guide future exploration. The protocol was registered at Figshare (https://doi.org/10.6084/m9.figshare.25399222.v1).

Eligibility criteria were defined using the Population‐Concept‐Context framework [18]: studies involving CYP (< 25 years) undergoing medical treatment for OS (ICCC VIII (a) Malignant Bone Tumours) [20] until completion of therapy. Studies with mixed cancer cohorts were eligible if OS comprised > 50% of the study cohort or was reported separately.

Initially, we included two concepts: (i) nutrition status at diagnosis and during treatment until 5 years post‐diagnosis and (ii) nutrition support interventions during cancer treatment (oral, enteral and/or parenteral) or individual nutrients given as part of current treatment protocols, for example, folinic acid. Due to the high volume and diversity of studies retrieved, we refined our focus to nutrition support interventions—an under‐researched but critical area for informing dietary support guidelines.

We excluded studies post‐treatment, other diagnoses, in‐vitro, animal or human studies investigating nutrients as novel treatments for OS. Studies solely linking height to OS incidence without addressing treatment‐related nutrition outcomes were excluded. We also excluded studies addressing nutrition‐related symptoms (e.g., nausea, vomiting, diarrhoea) unless they also included nutrition support, as this entailed inclusions relating to pharmacological management of treatment toxicity or evaluation of nutrition status rather than nutrition interventions. To maintain a focus on clinically applicable supportive care, complementary and alternative nutrition studies were also excluded.

All study designs were included except narrative and systematic reviews (although these were used for reference screening) and qualitative studies as beyond the scope of this review. Conference abstracts were included to capture the breadth of interest and minimise publication bias. No language or date restrictions were applied.

The Patient and Public Involvement Panel of the Bone Cancer Research Trust (https://www.bcrt.org.uk/) was consulted in March 2024 through focus groups, where the research questions' relevance and significance was discussed and endorsed and further involvement on study design invited. Feedback on protocol drafts has been provided by the panel, which helped determine that nutrition support rather nutrition status should be the focus of the review, due to its relevance to patient experience. Any publication will be shared with the panel and further dissemination discussed. The importance of this subject for those interviewed was highlighted by one respondent: ‘I felt a massive pressure to eat in order to keep my weight up so I could have treatment, which led to panic attacks (when the ward food trolley came round) because I just felt like I couldn't eat due to nausea/mouth ulcers/vomiting etc.’.

### 2.1 Search Strategy and Study Selection

We used a three‐step strategy (Supplementary Material: S1) to locate published and unpublished completed studies. First, a limited search of MEDLINE (OVID) and CINAHL (EBSCO) was conducted on 25th January 2024 using keyword terms in the title, abstract and subject heading fields related to osteosarcoma, paediatrics, nutritional support and nutritional status.

Second, a further 32 keywords—identified from relevant article titles, abstracts and indexing—were used to develop a full search in MEDLINE. Key articles known to meet the inclusion criteria were used to validate retrieval. The final search strategy was peer‐reviewed by a second medical librarian and adapted across all databases. The full searches were first completed on 1 May 2024 in MEDLINE (1945‐); EMBASE (1974‐); CINAHL (1981‐); EMCARE (1995‐); and Cochrane Central Register of Controlled Trials (CENTRAL). They were then re‐run for the period 1 May 2024–16 July 2025 (with nutrition status terms removed to improve relevance).

Third, the reference lists and citation indices of all included sources were screened for additional studies.

**Figure 1:**
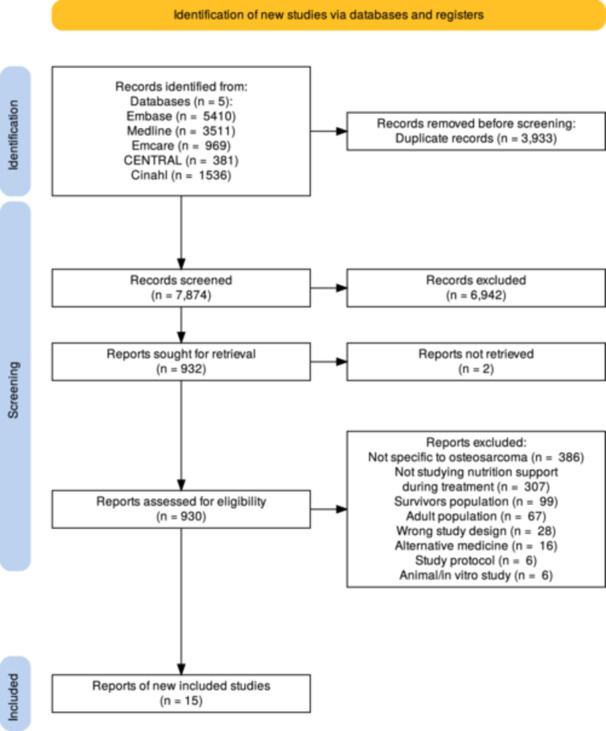
Preferred Reporting Items for Systematic Reviews and Meta‐Analyses (PRISMA) diagram for study screening and selection.

Citations were imported into Rayyan (https://new.rayyan.ai/) and duplicates removed. A pilot screening of 25 titles/abstracts was conducted independently by two reviewers (L. S. and M. S.). Following discussion and refinement of eligibility criteria, full screening commenced once 75% concordance was reached. L. S. and M. S. then screened all titles and abstracts. Full texts of potentially eligible sources were assessed by L. S., D. B. or R. R. I. Reasons for exclusion at full‐text stage were documented (Figure 1). Discrepancies were resolved through discussion. No new keywords emerged during screening.

### 2.2 Data Charting and Appraisal

L. S. charted data using a custom Excel tool (Supplementary Table: S2), capturing study design, objectives, population, methodology, intervention, outcomes and limitations. Following piloting, L. S. and R. R. I. reviewed and amended the tool to address missing or redundant inclusions. Data extraction was verified by R. R. I. Data were summarised descriptively in text and tables. JBI tools [18] were used to appraise full‐text, peer‐reviewed studies to identify methodological limitations. Based on the appraisal, studies were assigned a methodological quality rating for data synthesis.

## 3 Results

The full search (initial and re‐run combined) retrieved 7874 titles (Figure 1). Fifteen studies were included in data synthesis (Table 1). One study was published in French and machine translated [21].

**Table 1:** Characteristics of all studies on nutrition support for CYP undergoing OS treatment.

		Nutrition intervention type *n *= 15 studies	
Characteristic	Classification	Comprehensive nutrition (*n* = 10)	Micronutrient (*n* = 5)
Year	Before 2010	0	1
	2011–2015	3	1
	2016–2020	4	1
	2021–2025	3	2
Country income [22]	High income	8	2
	Middle income	2	3
	Low income	0	0
Publication format	Full publication	6	4
	Conference abstract	4	1
Study design	Prospective interventional	1	2
	Retrospective cohort	8	1
	Case study/series	1	2
Age^a^ (years)^b^	< 10	0	2
	10–16	7	2
	17–25	1	1
Nutrition support given	Early GT CNI tube feeds	5	0
	CNI oral nutrition supplements	2	0
	NG CNI tube feed	3	0
	PN	3	2
	CNI route (e.g, NG/PN) unspecified	2	N/A
	Appetite stimulants	1	0
	Thiamine	0	2
	Vitamin D	1	1
	Magnesium	0	1
	Mixed micronutrients (Mg sulphate, calcitriol, vitamin D3, calcium)	0	1

Abbreviations: CNI, comprehensive nutrition intervention; CYP, children and young people; GT, gastrostomy; Mg, magnesium; NG, nasogastric; OS, osteosarcoma; PN, parenteral nutrition.

^a^ Median, mean and actual age depending on cohort/case report.

^b^ Range of 0–18 years and range of ‘child to young adult’ reported by two sources.

### 3.1 Characteristics of All Nutrition Intervention Studies

Table 1 summarises characteristics of studies on nutrition interventions for CYP during OS treatment, with a combined OS sample size of 964. All originate from high‐ or middle‐income countries [22] (China, France, India, Indonesia, Japan, Mexico, United Kingdom (UK), United States of America (US)) published between 2001 and 2024. The median participant age was 13.3 years (IQR 2.2) aligning with typical diagnostic age. Ten sources (66%) described aspects of comprehensive nutrition interventions (CNI) addressing macro and micronutrient provision; five (33%) reported on individual micronutrients. Notably, all 10 CNI studies were published within the past 14 years, indicating growing research interest. The most common interventions were parenteral nutrition (PN) (5/15), and early enteral nutrition via gastrostomy (GT) (5/15).

Five sources were conference abstracts. These are included in Table 1 to illustrate the breadth of interest in the topic but were excluded from further synthesis and analysis due to limited detail and lack of peer review. A summary is provided in Supplementary Table: S3.

Findings from full individual studies regarding methodology, nutrition intervention characteristics, outcome measures, limitations and key results are detailed below, focussing on data for those with OS, where available.

### 3.2 Comprehensive Nutrition Intervention (CNI) Studies

Table 2 outlines six peer‐reviewed studies on CNI (median OS sample size: 24, range 1–59). Five used retrospective cohort designs; one US and three French studies evaluated local early systematic gastrostomy (GT) protocols in OS and mixed cancer cohorts, respectively. Two of these compared early GT to nasogastric (NG) feeding in response to weight loss across more than one centre [21, 23], and two evaluated primary radiological, or surgical GT placement [24]. One US cohort study explored types of nutrition interventions given to OS patients at one centre [25]. One [26] US case report described enteral nutrition via GT of an autistic child with OS, tracking their nutritional status throughout treatment.

**Table 2:** Characteristics, quality and key findings of peer‐reviewed sources reporting on comprehensive nutrition interventions for CYP undergoing OS treatment (total cohort data, stratified for OS where data available).

Study design and methodology rating	Sample size and sex	Age at diagnosis (years)	Diagnosis and treatment	Aim	Outcomes and methods	Key findings
Henry et al. [21] (France) Retrospective multi‐centre cohort Methodology rating: low^a^	*n* = 138 OS = 59 OS sex NR OS GT: *n* = 31 OS NG: *n* = 4 OS no GT/NG: *n* = 24	Mean ± SD eGT: 13 ± 3.8 NG: 11.4 ± 4 No eGT/NG: 11.9 ± 3.5 OS age NR	OS; EW Protocols: OS94 OS2006	Comparison of three NI groups: ‐ EN via eGT ‐ NG ‐ No eGT/NG	Nutritional status: BMI/Waterlow *Z*‐scores over time Frequency/type eGT complications Hospitalisations and duration for eGT/other complications Duration of PN with NI type Pre‐ and post‐op CT changes Time: pre‐op CT to surgery; surgery to post‐op CT Total treatment duration EFS at 4 years Medical record data	*BMI Z‐score changes:* Diagnosis—6/12 months: *p* = 0.09 At surgery: eGT −0.32; NG −0.70; No eGT/NG −0.34 (SD NR) 3 months after surgery: eGT −0.40; NG −0.65; No eGT/NG −0.30 (SD NR)
						*Complications:* ‐ eGT complication frequency: *n* = 37 (63.8%) ‐ OS eGT hospitalisations: *n* = 4; major *n* = 1; severe *n* = 3
						*Frequency OS GT vs. EW NG* (%) *p* = 0.02 OS eGT: *n* = 31 (53.4); EW NG: *n* = 16 [80] *Mean days on PN p* = 0.0038: eGT: 5.8 ± 14.2; NG: 9.9 ± 10.4; No eGT/NG: 8.5 ± 13.9 *EFS at 4 years: p* = 0.64
Schmitt et al. [23] (France) Retrospective multi‐centre cohort Methodology rating: low^a^	*N* = 48 OS = 31 eGT: Males = 15 No eGT: Males = 10 *p* = NR OS sex NR	Median (range) eGT: 13.3 (8.2–17.3) no eGT: 14.3 (9.3–18.9) OS NR	OS; EW Protocol OS 2006	Comparison of: eGT EN protocol vs. no systematic EN protocol (no eGT)	Nutrition status: Evolution of Wt/Ht and Wt/A *Z*‐scores during treatment and follow‐up Clinical status: Tx delay, duration GT CP frequency Cancer progression Data source NR	*% Wt change at 6 months* (mean ± SD): NS *p* NR: eGT: ‐0.1% ± 7.1%; no eGT: ‐4.7% ± 8.7% *Median Wt/Ht Z‐score at 6 months:* eGT: no ↓ (*p* NR). No eGT: ‐1.4 SD NS (*p* NR) *Post‐tumour resection CPs* NR *p* NR: eGT: *n* = 3; No eGT: *n* = 4. eGT local CPs: *n* = 27% *Relapse rate:* NS (*p* = 0.69). eGT: 31; No GT: 47
Richioud et al. [24] (France) Single‐centre cohort, follow‐up Methodology rating: moderate^b^	*n* = 11 OS = 6 Male *n* = 7 OS Male *n* = 3	Median (range): 13 (3–20) OS: 16 (10.5–20)	OS; UCN PA; NBL Protocol NR	Safety and effectiveness of primary RIG button placement	Nutrition status: Wt, Ht at diagnosis, at RIG insertion, 1, 2 and 3 months Incidence of RIG CPs Time from diagnosis to GT No. and type of CT pre‐ and post‐RIG insertion Medical record data	*1 month post‐RIG:* Wt↑: *n* = 7; Wt↓: *n* = 1. Maintained Wt: *n* = 2 *3 months post‐RIG:* Wt↑ *n* = 8; Wt ↓ *n* = 1 *Major RIG CPs: n* = 0. Minor CPs: *n* = 2 *Median (range) time from diagnosis to RIG:* 2 months (1–9) *No. CT cycles before RIG* (OS): 5.5 (2–11)
Dalton and Johnson [27] (USA) Single‐centre cohort, follow‐up Methodology rating: low^b^	*N* = 59 OS = 17 Sex NR	Age NR	NBL; OS; EW; RB; Wilms; HBL Protocol NR	Document incidence and risk factors for GT site infection‐related erythema in patients with solid tumours	% Wt↓ at diagnosis/relapse (NR) GT timing, type and location	*GT at diagnosis n* = 25 (43%); OS *n* = 16 (95%) vs. GT after Tx started *n* = 33 (57%); OS *n* = 1(5%) *GT placement location:* interventional radiology *n* = 7 (12%); theatre: *n* = 51 (88%) *GT type:* Corpak *n* = 3 (5%); Mic‐Key button *n* = 55 (95%) *GT erythema: n* = 31 (53%); OS GT erythema: *n* = 5 (29% of 17)
					Incidence of: – Likely infection‐related GT site erythema – Neutropenic erythema – Erythema‐related Tx delays	
					Use and type of antimicrobial prophylaxis Medical record data Descriptive statistics	
						*Of total erythema cases:* – GTs placed after Tx started: *n* = 23 (74%) – Neutropenic erythema: *n* = 22 (71%) – Erythema‐related therapy delay: *n* = 1 (3%) – Cases not given prophylactic abx *n* = 27 (87%)
Ringwald‐Smith et al. [25] (USA) Single‐centre cohort, follow‐up Methodology rating: moderate^b^	*n* = 48 OS = 48 Male *n* = 25	Median 13.7 (3.2–23.00)	OS Protocol OS99	Identify NI types given during treatment and effectiveness at improving weight status: NI types: AS, EN, PN	Ht, Wt and types of NI given at diagnosis, Week 12, 23, after EOT Age/sex with NI type Effectiveness of NIs to maintain/improve Wt: (transition between BMI categories) Clinical chart data	*Median (range) age (years) and NI* (*p* < 0.001): With NI: 9.9 (6.5–15.0). No NI: 15.5 (3.2–23.0). Sex NS. Ht/Wt NR *NI provided at any time points: n* = 14 *NI duration across all time points* (days); Median (range): 30 (1–30) *Effective NI: n* = 11; ineffective: *n* = 3 (*p* NR)^d^ *NI and BMI category (p* = *0.005):* NI given when UW *n* = 4. NI not given when: *n* = 0
Levin [26] (USA) Case report Methodology rating: high^c^	*N* = 1 Male	10	OS ASD Protocol AOST0331	Describe nutrition of child with ASD and OS	Nutrition status changes over treatment (BMI *Z*‐score for age/sex) Nutrition‐related signs and symptoms Data source NR	*GT insertion* </= 1 mo post‐diagnosis
						*BMI Z‐scores:* – Diagnosis: ‐1.63 – Pre‐surgery:‐ 0.41 SD – EOT: −0.54 (with limb amputation)
						Osteoporosis and vit D deficiency: 50,000 IU/wk × 8

Abbreviations: Abx, antibiotics; AS, appetite stimulants; ASD, autistic spectrum disorder; BMI, body mass index; CP, complications; CT, chemotherapy; eGT, early gastrostomy; EN, enteral nutrition; EFS, event‐free survival; EN, enteral nutrition; EOT, end of treatment; EW, Ewing sarcoma; F, female; Gp, group; GT, gastrostomy; HBL, hepatoblastoma; HT, height; IV, intravenous; IU, international units; M, male; Mg, magnesium; NBL, metastatic neuroblastoma; MRI, magnetic resonance imaging; MVI, multivitamin; NBL, neuroblastoma; NG, nasogastric; NI, nutrition intervention; No., number; NR, not reported; NS, not significant; OS, osteosarcoma; PA, pilocytic astrocytoma; PN, parenteral nutrition; RB, rhabdomyosarcoma; RIG, radiologically inserted gastrostomy; SD, standard deviation; TER, total energy requirements based on age, sex and weight; Tx, treatment; UCN, undifferentiated carcinoma of nasopharynx; UW, underweight; vit, vitamin; VS, versus; WE, Wernicke encephalopathy; Wl, week; WT, weight; Wt/Ht, weight‐for‐height; Wt/A, weight‐for‐age.

^a^ JBI Critical appraisal checklist for cohort studies.

^b^ JBI critical appraisal checklist for case series.

^c^ JBI critical appraisal checklist for case reports.

^d^ Unclear data. Reported as 7 and 7 in tabular data.

CNI has been characterised in two ways in the six studies:

3.3

*Route:* oral via appetite stimulants [25], enteral via NG or GT [21, 23, 24, 26, 27], and parenteral nutrition [21, 25]. No studies reported the use of oral nutrition supplements.

3.4

*Timing:* early enteral support placement (1–3 months before surgery) either through individual nutrition status assessment [26] or protocol‐driven [21, 23, 24]. Indication for placement is not reported by one study where 95% of patients received a GT at diagnosis [27].

Where available, data on the nutritional delivery route, regimen and content have been summarised in Table 3. Only the case report detailed nutritional regimens (peptide, 1 kcal/mL feed given day/night via pump contributing 50%–100% requirements) [26]. Two other studies [23, 24] reported GT feed delivery via pump, nocturnally and/or daytime with less detail.

**Table 3:** Summary of comprehensive and micronutrient nutrition intervention routes, content and regimens.

Authors	Nutrition delivery route	Nutritional product, content and regimen
*Comprehensive nutrition intervention studies*		
Henry et al. [21]	EN via eGT, EN via NG No eGT/NG	Not reported
Schmitt et al. [23]	EN via eGT	Feed given overnight ± daytime bolus Ad libitum oral diet Volume adapted to tolerance Feed product/content not reported
Richioud et al. [24]	EN via RIG	EN feed volume adapted to needs, ↑ over 5 days to 100% as cyclized injections (10 h/day) Feed product/content not reported
Dalton and Johnson [27]	EN via GT	Not reported
Ringwald‐Smith et al. [25]	Appetite stimulants, EN, PN	Dronabinol, cyproheptadine, megestrol (dose not reported) Not reported for EN and PN
Levin [26]	EN via eGT	1 Kcal/mL peptide feed through Tx and beyond. Night + daytime pumped boluses (50%–100% TER); ad libitum oral diet.
*Micronutrient intervention studies*		
Lee et al. [28]	PN + thiamine	Not reported for PN 1000 mg and 1500 mg thiamine daily to each patient, respectively (route and duration not reported)
Perko et al. [29]	PN + IV thiamine	Not reported
Castelan‐Martinez et al. [30]	Oral	Mg 250 mg/day at discharge (end date not reported) Control group: no Mg
Nozaki et al. [31]	Oral	Oral aD3 (0.75–4 µg) for mean 25 months (SD not reported) Oral aD3 > 1500 µg total dose Oral aD3 4 µg daily > 6 months

Abbreviations: aD3, active vitamin D3; eGT, early gastrostomy; EN, enteral nutrition; IV, intravenous; Mg, magnesium; NG, nasogastric; PN, parenteral nutrition; RIG, radiologically inserted gastrostomy; TER, total energy requirement; Tx, treatment.

Primary outcomes for 5 of 6 CNI studies were anthropometric status (weight‐for‐height *Z*‐scores) recorded at diagnosis and regular (but variable) intervals: (1, 2, 3, 6 months, pre‐/post‐surgery, end of treatment). One study [25] assessed transitions between BMI percentile‐based categories to evaluate nutrition intervention effectiveness. Gastrostomy safety was the second most reported outcome (4 of 6 sources), categorised by author‐defined morbidity [21, 23, 24, 27]. Other outcomes included event‐free survival [21], relapse and mortality rate [23], tumour resection complications [23] and chemotherapy modifications due to weight changes [21].

Henry et al. [21] found OS patients more likely to receive GT than NG, likely due to protocol. Early GT/NG support showed a trend towards BMI *Z*‐score maintenance versus no early support (*p* = 0.09) and GT was linked to fewer days on PN (average 5.8 days vs. 10.4 with NG (*p* ≤ 0.004)). Similarly, Schmitt et al. [23] reported non‐significant weight loss reduction (mean 0.1% vs. 4.7%) and weight‐for‐height *Z*‐score stability with early GT versus −1.4 SD change at 6 months without an early GT (*p* not reported).

Henry et al. [21] reported GT complications in 37 (64%) of patients, mostly minor, with 4 requiring hospitalisation. Schmitt reported 27 local GT complications in 29 patients. Dalton and Johnson [27] reported GT‐site erythema concerning for infection in 5 (29%) OS patients. These three studies lacked complete reporting and validated outcome measures, contributing to a low‐quality rating. Richioud et al. [24] observed that 8 of 11 patients gained weight 3 months after radiologically inserted GT (RIG) placement, with 2 minor complications. In the sole OS‐only CNI cohort, Ringwald‐Smith et al. [25] found 14 of 48 patients received nutrition support, with younger age significantly associated with intervention (median 9.9 years vs. 15.5 years (*p* < 0.001)). These two studies were rated moderate quality but conducted at single centres.

See Supplementary Tables: S4 for complete outcome data, appraisal and findings summaries.

### 3.5 Micronutrient Intervention Studies

Table 4 summarises four published studies on micronutrient interventions during OS treatment (median *n* = 16; range 1–63).

**Table 4:** Characteristics, quality and key findings of peer‐reviewed sources of evidence reporting on micronutrient support for CYP undergoing OS treatment (total cohort data, stratified for OS where data available).

Study design and methodology rating	Sample size, sex and age (years)	Diagnoses and OS treatment protocol	Study aims	Outcomes and findings
Lee et al. [28] (China) Case series Methodology rating: moderate^a^	*n* = 3 OS *n* = 2 (1M, 1F) Age: 12; 17	OS; HUS Protocol NR	Raise awareness of paediatric (WE) by describing local cases	*OS presenting symptoms:* confusion, athetosis *n* = 1; Dullness, flaccid tone *n* = 1 *OS PN duration pre‐onset:* 1 week: *n* = 1; 2 months: *n* = 1 *OS MRI:* Dorsomedial thalamic T2 hyperintensity and restricted diffusion *n* = 2 *OS lab results post‐Th:* Normal/↑Transketolase: *n* = 2; Normal Th diphosphate *n* = 2 *OS clinical outcome:* complete symptom resolution *n* = 1; Improved mental status but limited motor control *n* = 1
Perko et al. [29] (USA) Case series Methodology rating: low^a^	*n* = 5 OS *n* = 1 (F) Age: 9	MBL; OS; AML; RB Protocol NR	Highlight risk of WE during IV MVI shortages. Need for early diagnosis	*OS clinical status* by MRI *brain 7 days post Th and PN:* mixed response (↓T2 prolongation of subthalamic nuclei/mammillary bodies, ↑T2 prolongation of bilateral caudate heads). Gradual neurological improvement to baseline.
Castelan‐Martinez et al. [30] (México) Randomised controlled trial Methodology rating: high^b^	*n* = 46 OS = 63/101 CT cycles *F* = 62 CT cycles Median age (IQR): 13.5 (3.6)	OS; GCT; HBL; EP Protocol NR	Assess efficacy and safety of oral Mg to reduce FN, hypoMg and septic shock in paediatric patients given CIS	*Incidence of FN* (*n*): Mg Gp = 14; Control = 27; RR (95% CI) = 0.53 (0.32–0.89) (*p* = 0.019) *Incidence of hypoMg* (*n*): Mg Gp = 7; Control = 10; RR = 0.71 (0.3–1.73) *Incidence of septic shock* (*n*): Mg Gp = 5; Control = 14; RR = 0.43 (0.02–0.94)
Nozaki et al. [31] (Japan) Randomised controlled trial Methodology rating: low^b^	*n* = 29 (all OS) aD3 Gp: *n* = 15M; 3F Control: *N* = 5M; 6F Median age (range): 19 (9–58)	OS protocol NR. Included surgery + pre‐ and post‐DOX + CIS	Survival OS patients given oral aD3 vs. without oral aD3	*5‐year survival rate* (%): (*p* NR): aD3 group = 61.1; Control = 63.6 *10‐year survival rate:* NS (*p* = 0.3823): aD3 group = 61.1; Control = 33.9 *Median (range) survival time* (months) (*p* NR): aD3 group = 88 (17–155); Control = 38 (6–180)

Abbreviations: aD3, active vitamin D3; AML, acute myeloid leukaemia; CIS, cisplatin; CT, chemotherapy; CYP, children and young people; DOX, doxorubicin; EP, ependymoma F, female; FN, febrile neutropenia; GCT, germ cell tumour; Gp, group; HBL, hepatoblastoma; HUS, haemolytic uraemic syndrome; IV, intravenous; M, male; Mg, magnesium; OS, osteosarcoma; MBL, medulloblastoma; MRI, magnetic resonance imaging; MVI, multivitamin; NR, not reported; NS, not significant; OS, osteosarcoma; RB, rhabdomyosarcoma; Th, thiamine; PN, parenteral nutrition; WE, Wernicke's encephalopathy.

^a^ JBI critical appraisal checklist for case series.

^b^ JBI Critical appraisal tool for assessment of risk of bias for RCTs.

Two studies were randomised, controlled trials: Castelan‐Martinez et al. [30] provided 250 mg/day oral magnesium at discharge from admission for cisplatin delivery until Day 1 of febrile neutropenia (FN). They measured the incidence of FN, hypomagnesemia and septic shock. The well‐designed trial used true randomisation, adjustment for baseline variables and validated tracking of compliance. Magnesium supplementation significantly reduced FN incidence: Mg group = 14; Control = 27; RR (95% CI) = 0.53 (0.32–0.89) (*p* = 0.019), suggesting need for further exploration with larger, blinded trials.

Nozaki et al. [31] investigated two dosing strategies of active vitamin D3 assessing 5‐ and 10‐year survival. They [31] also identify the need for larger, multicentre trials for better statistical power. However, incomplete reporting of baseline clinical, nutritional and sociodemographic data and the randomisation method weakens the conclusion that the small sample size explains a lack of effect. This was the only micronutrient study focussing solely on OS, which found no differences in outcomes for supplementation.

Two case series [28, 29] described thiamine deficiency treatment in three patients, using laboratory and MRI‐based recovery measures [29]. Lee et al. reported 1000–1500 mg thiamine given daily (route and duration unknown) for Wernicke's encephalopathy associated with osteosarcoma [28] in two patients receiving PN (for 1 week and 2 months) without micronutrient content description. Perko et al. [29] reported one case receiving intravenous thiamine and total PN following low thiamine levels and MRI findings. Methodological quality was weak to moderate due to incomplete reporting of nutrition interventions (Table 3), baseline status and population description.

For full findings, limitations and critical appraisal of studies, see Supplementary Tables: S4.

## 4 Discussion

This review mapped research on nutrition support interventions for CYP undergoing treatment for OS, to establish methodologies, interventions, outcomes and limitations to inform research priorities to guide clinical nutrition care.

We found peer‐reviewed studies are recent, from high‐ or middle‐income countries, mostly involving children over 10 years, and feature small, often mixed cohorts. Most retrospectively examine tube feeding, or prospectively trial micronutrient supplementation. Otherwise, they report anecdotal thiamine intervention for deficiency. Common outcomes include anthropometric nutritional status, intervention‐related complications (e.g., gastrostomy) or clinical markers including survival.

An emerging theme is the method of tube feeding and timing of initiation, particularly early/prophylactic GT versus reactive NG feeding [21, 23]. While one US study reported 95% GT insertion at diagnosis in an OS cohort [27], comparison of this practice with reactive tube feeding for bone tumours (including OS) is currently unique to France, where systematic GT insertion is integrated into local protocols based on nutritional status [24] or diagnosis [21, 23]. While statistically significant benefits were not demonstrated, trends suggest nutritional stability with early GT. Supportive evidence from other paediatric cancer cohorts [32, 33, 34, 35] includes a multicentre, retrospective study in patients with medulloblastoma (ITR‐3 Rating 3) [34]. Results revealed 5.9% less weight loss (95% CI: 2.1%–9.7% *p* = 0.02) with early GT versus reactive NG‐predominant feeding, for a treatment associated with high rates of constipation and vomiting toxicity. Nutritional status improvement through early tube feeding (within the first 2 weeks of treatment) has also been associated with fewer non‐leukopenic infections in a mixed cohort including OS [36], suggesting nutritional therapy may alleviate clinical morbidity.

Despite evidence demonstrating the clinical benefits of enteral feeding, concerns persist regarding GT infections in this vulnerable population. However, existing literature [37] and studies included in this review [21, 23, 24, 26, 27] indicate these are typically minor and may relate to the timing of placement. In contrast, no studies collected data on NG‐related complications such as emesis frequency, dislodgement, re‐insertion delays or the potentially traumatic experience of NG placement [38]. These events are likely under‐recorded in clinical notes, contributing to reporting bias amongst the retrospective included studies. Addressing these gaps is essential for a more comprehensive and balanced comparison of feeding interventions. Indeed a prospective cohort study comparing complications of prophylactic GT versus NG support for paediatric bone marrow transplantation showed that of 19 children with NG tubes, the tube was either pulled out, vomited or accidentally dislodged 109 times over the period of the study [35].

Collectively these studies highlight the need to map global practices similar to those reported by Henry et al. [21], Schmitt et al. [23], Richioud et al. [24] and Dalton and Johnson [27] for OS. Systematic documentation of institutional protocols would enable rigorous evaluation of efficacy and safety in OS, and other high nutrition risk treatments. No study has compared these approaches specifically for OS, without other cancers or treatment bias, likely due to limited sample sizes and funding [23]—persistent challenges for paediatric oncology nutrition research.

Progress for rare diseases such as OS depends on access to national (or larger) data sets, pooled electronic healthcare systems [39], informatics and data analyst collaboration and harmonised nutritional diagnosis and care terminology. It is important that registries such as the Childhood Cancer Data Initiative [40] include nutrition data to support this. The EPICkids protocol [41] is establishing a nutrition biobank for children with acute lymphoblastic leukaemia and brain tumours. It collects detailed dietary, anthropometric, lifestyle, sociodemographic and biological sample data throughout treatment, to assess growth, micronutrient status, metabolism, gut microbiome and treatment outcomes. Its prospective design, and inclusion of validated assessment tools, sets it apart from the CNI‐related studies in this review, where only one refers to a peer‐reviewed tool for nutrition support complication categorisation [24]. Development of core outcome sets for nutrition status and support complications, recently undertaken in adults [42], could also enhance data quality and comparability and has been identified as a priority [13]. Combined with prospective, multicentre designs, as with EPICKids, this could transform nutrition research and care for OS.

All CNI sources assess weight‐based outcomes, reflecting clinical norms [1]. Whilst BMI fluctuates considerably during and beyond treatment for OS [43], weight is a limited nutrition indicator for patients with solid tumours [44], particularly for OS [45]. Studies [21, 25] acknowledge multiple factors that complicate interpretation: hyperhydration, alterations in skeletal muscle and fat mass due to the tumour, its treatment, and reduced physical activity, as well as surgical amputations. Routine clinical incorporation of informative, non‐invasive and pragmatic tools such as bioelectrical impedance, skinfold thicknesses and mid‐upper arm circumference, with validated reference data [44], could enhance clinical monitoring and research quality—particularly during the pubertal peak age of OS, where body composition changes rapidly. Accurately identifying those most nutritionally depleted enables targeted, proactive nutrition support where it is most needed, and enhances understanding of treatment‐related malnutrition.

A key gap across the reviewed studies is the limited reporting on nutritional intervention content, including enteral feed type, volume, intake, target requirements, feeding regimens, gastrointestinal tolerance and PN composition. This gap is notable given concerns of thiamine deficiency [28, 29], highlighting the need for precise nutrient monitoring. Only one case report [26] provides comprehensive data on nutritional interventions; such information is essential for tailoring feeding strategies for children with fluctuating appetites, requirements and gastrointestinal function [36].

Hydrolysed protein feeds are commonly used to enhance enteral tolerance during mucositis‐related vomiting [46] (including for OS) [47], although evidence from randomised controlled trials in paediatric oncology remains scarce [13, 48]. Indeed, Meyer and colleagues found mixed effects of protein hydrolysis on gastric emptying rate in non‐oncology paediatric populations [49], identifying a need for further comparisons with similar feed ingredients in populations who might benefit. This is important where such enteral feeds, often very low in fibre, can provide sustained, but monotonous, nutrition for the gut microbiota of children already adversely affected by cancer treatment, and where maintenance of a healthy, diverse composition has implications for infection risk [50]. Interest in blended food tube feeds is growing, partly due to improved tolerance and diverse fibre composition [51]. Their potential benefits in paediatric oncology remain promising but largely unstudied, possibly in part due to the predominant use of NG tubes. Blended feed administration is recommended via a larger‐bore GT tube [52], highlighting the careful consideration required when selecting the nutrition support route. The microbial quality and safety of such feeds have also not been confirmed [53], which is of particular significance in this immunologically vulnerable group. Successful use of commercial feeds that incorporate food‐derived ingredients has recently been documented in paediatric oncology patients [54].

This review also highlighted research activity outside peer‐reviewed journals, with 4 of 10 CNI studies sourced solely from conference abstracts—indicating substantial interest but possibly limited resource or full publication opportunities. None reported funding and all originated from lower‐middle‐income countries, or dietetic teams in high‐income settings. These studies had a combined sample size far exceeding the peer‐reviewed selection (*n* = 643 vs. *n* = 162) suggesting strong potential for high‐impact findings, if adequately supported. Historically, allied health research has lagged behind medical and nursing fields, but this is changing [55]. The rise in CNI studies since 2010 (Table 1) and recent international collaborations [14], reflect a global recognition of the need to develop evidence‐based recommendations for clinical nutrition practice [14].

Exploration of the personal experiences of nutritional approaches for young people with OS was outside this review's scope but included studies recognise this [21, 23] as an important avenue for further research. Any effective clinical application of quantitative nutrition research amongst service users must include consideration of the receiver's experience, and their informational and decision‐making needs [15] and incorporate their priorities into the chosen outcome measures [13]. This is particularly important for adolescents who may seek greater autonomy in healthcare decisions [16], and for an intervention where they may be significant aesthetic and comfort‐related implications. This is strongly supported by a recent qualitative study of children undergoing allogeneic bone marrow transplantation, which found children wanted to be involved in choice of feeding tubes and decision‐making [56]. Evidence also suggests younger patients receive more nutrition support in mixed [32] and OS cohorts [25], underscoring the need to understand age‐related preferences.

### 4.1 Limitations of Scoping Review Process

We did not explore all ‘grey’ literature repositories, for example, CORE, eTHOS, OpenGrey, Mednar or Google Scholar, so papers meeting inclusion criteria may have been missed. Screening was affected by author changes between title/abstract and full‐text phases, potentially affecting final selection, although each stage involved a comprehensive strategy and at least two independent reviewers. One paper was machine translated, which may have affected nuanced interpretation. Risk of bias tools were used for appraisal, however, assessments remain at risk of subjectivity.

## 5 Conclusion

Nutrition intervention studies for OS primarily focus on comprehensive nutrition support during treatment, with some exploring specific micronutrients (thiamine, magnesium and vitamin D). Interest is growing in researching proactive GT‐based support strategies and their effect on nutritional status and safety. Prospective, randomised studies with larger sample sizes of purely OS cohorts, full characterisation of the nutrition received, non‐weight dependent nutritional status and validated clinical outcome measures are needed to generate high‐quality evidence. Incorporating a qualitative exploration of survivors' lived experiences of these interventions is also needed to fully consider their value and application. Therefore, a mixed‐methods approach, similar to that already undertaken during allogeneic bone marrow transplantation [56], incorporating both strands of enquiry would complement and support the generation of a stronger evidence base around this under‐researched topic.

## Author Contributions

**Laura Sealy:** concept, stakeholder consultation, protocol development, search strategy development, study screening and selection, data charting, analysis, report writing and reviewing. **Maddie Smith:** study screening and selection, report reviewing and editing. **Sarah Barrett:** search strategy development and search implementation, report reviewing and editing. **Divyalakshmi Bhaskaran:** Study screening andselection, report reviewing and editing. **Helen Rees:** protocol development, report reviewing and editing. **Raquel Revuelta Iniesta:** guarantor, protocol review, study screening and selection, data charting verification, data analysis, report reviewing and editing.

## Ethics Statement

The authors have nothing to report.

## Conflicts of Interest

All authors have completed the ICMJE uniform disclosure form at http://www.icmje.org/disclosure-of-interest/ and declare: no support from any commercial organisation for the submitted work; no financial relationships with any organisations that might have an interest in the submitted work in the previous 3 years; no other relationships or activities that could appear to have influenced the submitted work.

## Supporting information


**Supplemental Material S1:** Full search strategy.

**Supplemental Table S2:** Data charting form template.

**Supplemental Table S3:** Conference abstracts meeting inclusion criteria but excluded at full text characterization.

**Supplemental Table S4:** Full table of key findings, limitations and critical appraisal of included full text sources.
