# Supplementary material for: Nutrition Support Interventions for Children and Young People Treated for Osteosarcoma: A Scoping Review
Source: J Hum Nutr Diet. 2025 Nov 28;38(6):e70172. doi: 10.1111/jhn.70172 (PMC12661479; doi:10.1111/jhn.70172)
Supplement: Supplementary file 1 — Supplemental Material S1: Full search strategy. [file JHN-38-0-s003.docx]

**Search Results**

| **Database Name** | **Platform** | **Date Coverage*** | **Date of Search** | **Results** |
| --- | --- | --- | --- | --- |
| 1.Embase | Ovid | 1974-present | 1 May 2024  16 July 2025 | 5004  406 |
| 2. Medline | Ovid | 1945-present | 1 May 2024  16 July 2025 | 3384  127 |
| 3.Emcare | Ovid | 1981-present | 1 May 2024  16 July 2025 | 896  73 |
| 4.CENTRAL | Cochrane |  | 1 May 2024  16 July 2025 | 357  24 |
| 5. Cinahl | Ebsco | 1981-present | 1 May 2024  16 July 2025 | 1462  74 |

**Search Strategies**

**Ovid EMBASE**

1 exp osteosarcoma/
 2 (osteosarcoma* or "osteogenic sarcoma*").mp.
 3 bone sarcoma/
 4 ("bone tumo?r*" or "bone sarcoma*").ti,ab,kw.
 5 "solid tumo?r*".ti,ab.
 6 ((child* or p?ediatric or adolescen* or young) adj3 (cancer or oncology)).ti.
 7 or/1-6
 8 child/
 9 adolescent/
 10 pediatrics/
 11 young adult/
 12 (child* or children* or p?ediatric* or young or adolescen* or teen* or "TYA" or "YA" or school* or girl* or boy* or infan* or juvenile* or youth*).ti,ab,kw.
 13 or/8-12
 14 dual energy x ray absorptiometry/
 15 childhood obesity/
 16 body composition/
 17 exp nutritional status/
 18 exp nutritional disorder/
 19 exp nutritional assessment/
 20 anthropometry/
 21 ("DEXA" or "DXA" or "dual-energy x-ray absorptiometry" or "dual energy x ray absorptiometry").ti,ab,kw.
 22 ("body composition" or "body weight" or "body size" or "body fat" or "skinfold thickness" or "skin-fold thickness" or "skin fold thickness" or anthropometr* or "mid-upper arm circumference" or "mid upper arm circumference").ti,ab,kw.
 23 ("body mass index" or "BMI").ti,kw.
 24 ((weight or height) adj ("z score" or z-score or percentile*)).ti,ab,kw.
 25 ("weight gain" or "weight loss").ti,ab,kw.
 26 ("lean body mass" or "fat-free mass" or "fat free mass" or "skeletal muscle" or "muscle mass" or sarcopenia).ti,ab,kw.
 27 (malnutrition or malnourished or undernutrition or overnutrition or "nutrition* assessment*").ti,ab,kw.
 28 ((antioxidant* or vitamin* or mineral* or micronutrient* or "trace element*" or electrolyte* or glucose or glutamine or "amino acid*") adj2 (deficien* or status)).ti,ab,kw.
 29 (appetite or "energy intake" or "energy expenditure").ti,ab,kw.
 30 basal metabolic rate/
 31 ("bio-electric* impedance" or "bioelectric* impedance").ti,ab,kw.
 32 bioelectrical impedance analysis/
 33 or/14-32
 34 exp nutritional support/
 35 enteric feeding/
 36 exp parenteral nutrition/
 37 nutrition policy/
 38 diet therapy/
 39 dietetics/
 40 diet/
 41 gastrostomy/
 42 digestive tract intubation/
 43 ("tube feed*" or "nasogastric tube*" or gastrostom*).ti,ab,kw.
 44 ((nasoenteral or enteral or parenteral) adj2 (nutrition or feed*)).ti,ab,kw.
 45 fortified food/
 46 dietary supplement/
 47 electrolyte/ad, dt [Drug Administration, Drug Therapy]
 48 glucose/ad, dt [Drug Administration, Drug Therapy]
 49 amino acid/ad, dt [Drug Administration, Drug Therapy]
 50 trace element/ad, dt [Drug Administration, Drug Therapy]
 51 protein intake/
 52 intestine flora/
 53 (nutrition* or nutrient* or diet*).ti,ab,kw.
 54 ("oral supplement*" or formula? or (food* adj fortifi*)).ti,ab,kw.
 55 ((antioxidant* or mineral* or micronutrient* or "trace element*" or vitamin* or electrolyte* or glucose or glutamine or "amino acid*") adj2 (intake or therap* or supplement*)).ti,ab,kw.
 56 "protein intake".ti,ab,kw.
 57 (microbiome or "gut biome" or "gut microbiota").ti,ab,kw.
 58 or/34-57
 59 7 and 13 and 33
 60 7 and 13 and 58
 61 59 or 60

Update search run as:

62 7 and 13 and 58
63 limit 62 to dc=20240501-20250716

**Ovid MEDLINE(R) ALL**

1 exp Osteosarcoma/
 2 (osteosarcoma* or "osteogenic sarcoma*").mp.
 3 Bone Neoplasms/
 4 ("bone tumo?r*" or "bone sarcoma*").ti,ab,kw.
 5 "solid tumo?r*".ti,ab.
 6 ((child* or p?ediatric or adolescen* or young) adj3 (cancer or oncology)).ti.
 7 or/1-6
 8 Child/
 9 Adolescent/
 10 Pediatrics/
 11 Young Adult/
 12 (child* or children* or p?ediatric* or young or adolescen* or teen* or "TYA" or "YA" or school* or girl* or boy* or infan* or juvenile* or youth*).ti,ab,kw.
 13 or/8-12
 14 Absorptiometry, Photon/
 15 Pediatric Obesity/
 16 exp "Body Weights and Measures"/
 17 exp Body Composition/
 18 exp Nutritional Status/
 19 exp Nutrition Disorders/
 20 exp Nutrition Assessment/
 21 Anthropometry/
 22 ("DEXA" or "DXA" or "dual-energy x-ray absorptiometry" or "dual energy x ray absorptiometry").ti,ab,kw.
 23 ("body composition" or "body weight" or "body size" or "body fat" or "skinfold thickness" or "skin-fold thickness" or "skin fold thickness" or anthropometr* or "mid-upper arm circumference" or "mid upper arm circumference").ti,ab,kw.
 24 ("body mass index" or "BMI").ti,kw.
 25 ((weight or height) adj ("z score" or z-score or percentile*)).ti,ab,kw.
 26 ("weight gain" or "weight loss").ti,ab,kw.
 27 ("lean body mass" or "fat-free mass" or "fat free mass" or "skeletal muscle" or "muscle mass" or sarcopenia).ti,ab,kw.
 28 (malnutrition or malnourished or undernutrition or overnutrition or "nutrition* assessment*").ti,ab,kw.
 29 ((antioxidant* or vitamin* or mineral* or micronutrient* or "trace element*" or electrolyte* or glucose or glutamine or "amino acid*") adj2 (deficien* or status)).ti,ab,kw.
 30 (appetite or "energy intake" or "energy expenditure").ti,ab,kw.
 31 Basal Metabolism/
 32 ("bio-electric* impedance" or "bioelectric* impedance").ti,ab,kw.
 33 Electric Impedance/
 34 or/14-33
 35 exp Nutritional Support/
 36 exp Enteral Nutrition/
 37 exp Parenteral Nutrition/
 38 exp Nutrition Policy/
 39 exp Nutrition Therapy/
 40 exp Diet Therapy/
 41 exp Dietetics/
 42 exp Diet/
 43 exp Gastrostomy/
 44 Intubation, Gastrointestinal/
 45 ("tube feed*" or "nasogastric tube*" or gastrostom*).ti,ab,kw.
 46 ((nasoenteral or enteral or parenteral) adj2 (nutrition or feed*)).ti,ab,kw.
 47 Food, Fortified/
 48 Dietary Supplements/
 49 Electrolytes/ad, tu [Administration & Dosage, Therapeutic Use]
 50 Glucose/ad, tu [Administration & Dosage, Therapeutic Use]
 51 exp Amino Acids/ad, tu [Administration & Dosage, Therapeutic Use]
 52 Trace Elements/ad, tu [Administration & Dosage, Therapeutic Use]
 53 exp Micronutrients/
 54 exp Dietary Proteins/
 55 Gastrointestinal Microbiome/
 56 (nutrition* or nutrient* or diet*).ti,ab,kw.
 57 ("oral supplement*" or formula? or (food* adj fortifi*)).ti,ab,kw.
 58 ((antioxidant* or mineral* or micronutrient* or "trace element*" or vitamin* or electrolyte* or glucose or glutamine or "amino acid*") adj2 (intake or therap* or supplement*)).ti,ab,kw.
 59 "protein intake".ti,ab,kw.
 60 (microbiome or "gut biome" or "gut microbiota").ti,ab,kw.
 61 or/35-60
 62 7 and 13 and 34
 63 7 and 13 and 61
 64 62 or 63

Update search run as:

65 7 and 13 and 61

66 limit 65 to dt=20240501-20250716

**Ovid Emcare**

1 exp osteosarcoma/
 2 (osteosarcoma* or "osteogenic sarcoma*").mp.
 3 bone tumor/
 4 ("bone tumo?r*" or "bone sarcoma*").ti,ab,kw.
 5 "solid tumo?r*".ti,ab.
 6 ((child* or p?ediatric or adolescen* or young) adj3 (cancer or oncology)).ti.
 7 or/1-6
 8 child/
 9 adolescent/
 10 pediatrics/
 11 young adult/
 12 (child* or children* or p?ediatric* or young or adolescen* or teen* or "TYA" or "YA" or school* or girl* or boy* or infan* or juvenile* or youth*).ti,ab,kw.
 13 or/8-12
 14 dual energy x ray absorptiometry/
 15 childhood obesity/
 16 body composition/
 17 exp nutritional status/
 18 exp nutritional disorder/
 19 nutritional assessment/
 20 anthropometry/
 21 ("DEXA" or "DXA" or "dual-energy x-ray absorptiometry" or "dual energy x ray absorptiometry").ti,ab,kw.
 22 ("body composition" or "body weight" or "body size" or "body fat" or "skinfold thickness" or "skin-fold thickness" or "skin fold thickness" or anthropometr* or "mid-upper arm circumference" or "mid upper arm circumference").ti,ab,kw.
 23 ("body mass index" or "BMI").ti,kw.
 24 ((weight or height) adj ("z score" or z-score or percentile*)).ti,ab,kw.
 25 ("weight gain" or "weight loss").ti,ab,kw.
 26 ("lean body mass" or "fat-free mass" or "fat free mass" or "skeletal muscle" or "muscle mass" or sarcopenia).ti,ab,kw.
 27 (malnutrition or malnourished or undernutrition or overnutrition or "nutrition* assessment*").ti,ab,kw.
 28 ((antioxidant* or vitamin* or mineral* or micronutrient* or "trace element*" or electrolyte* or glucose or glutamine or "amino acid*") adj2 (deficien* or status)).ti,ab,kw.
 29 (appetite or "energy intake" or "energy expenditure").ti,ab,kw.
 30 basal metabolic rate/
 31 ("bio-electric* impedance" or "bioelectric* impedance").ti,ab,kw.
 32 impedance/
 33 or/14-32
 34 exp nutritional support/
 35 enteric feeding/
 36 exp parenteral nutrition/
 37 nutrition policy/
 38 diet therapy/
 39 dietetics/
 40 diet/
 41 gastrostomy/
 42 digestive tract intubation/
 43 ("tube feed*" or "nasogastric tube*" or gastrostom*).ti,ab,kw.
 44 ((nasoenteral or enteral or parenteral) adj2 (nutrition or feed*)).ti,ab,kw.
 45 fortified food/
 46 dietary supplement/
 47 electrolyte/
 48 amino acid/
 49 trace element/
 50 protein intake/
 51 intestine flora/
 52 (nutrition* or nutrient* or diet*).ti,ab,kw.
 53 ("oral supplement*" or formula? or (food* adj fortifi*)).ti,ab,kw.
 54 ((antioxidant* or mineral* or micronutrient* or "trace element*" or vitamin* or electrolyte* or glucose or glutamine or "amino acid*") adj2 (intake or therap* or supplement*)).ti,ab,kw.
 55 "protein intake".ti,ab,kw.
 56 (microbiome or "gut biome" or "gut microbiota").ti,ab,kw.
 57 or/34-56
 58 7 and 13 and 33
 59 7 and 13 and 57
 60 58 or 59

Update search run as:

61 7 and 13 and 57

62 limit 61 to dc=20240501-20250716

**Cochrane Central Register of Controlled Trials (CENTRAL)**

 #1 MeSH descriptor: [Osteosarcoma] explode all trees
 #2 (osteosarcoma* or (osteogenic NEXT sarcoma*))
 #3 MeSH descriptor: [Bone Neoplasms] this term only
 #4 ((bone NEXT tumo?r*) OR (bone NEXT sarcoma*)):ti,ab,kw
 #5 (solid NEXT tumo?r*):ti,ab
 #6 ((child* OR p?ediatric OR adolescen* OR young) NEAR/3 (cancer or oncology)):ti
 #7 (#1 or #2 or #3 or #4 or #5 or #6)
 #8 MeSH descriptor: [Child] this term only
 #9 MeSH descriptor: [Adolescent] this term only
 #10 MeSH descriptor: [Pediatrics] this term only
 #11 MeSH descriptor: [Young Adult] this term only
 #12 (child* or children* or p?ediatric* or young or adolescen* or teen* or "TYA" or "YA" or school* or girl* or boy* or infan* or juvenile* or youth*):ti,ab,kw
 #13 (#8 OR #9 OR #10 OR #11 OR #12)
 #14 MeSH descriptor: [Absorptiometry, Photon] this term only
 #15 MeSH descriptor: [Pediatric Obesity] this term only
 #16 MeSH descriptor: [Body Composition] explode all trees
 #17 MeSH descriptor: [Nutritional Status] explode all trees
 #18 MeSH descriptor: [Nutritional Status] explode all trees
 #19 MeSH descriptor: [Nutrition Disorders] explode all trees
 #20 MeSH descriptor: [Nutrition Assessment] explode all trees
 #21 MeSH descriptor: [Anthropometry] this term only
 #22 ("DEXA" OR "DXA" OR "dual-energy x-ray absorptiometry" or "dual energy x ray absorptiometry"):ti,ab,kw
 #23 ("body composition" OR "body weight" OR "body size" OR "body fat" OR "skinfold thickness" OR "skin-fold thickness" OR "skin fold thickness" OR anthropometr* OR "mid-upper arm circumference" OR "mid upper arm circumference"):ti,ab,kw
 #24 ("body mass index" or "BMI"):ti,kw
 #25 ((weight OR height) NEAR/1 ("z score" OR z-score OR percentile*)):ti,ab,kw
 #26 ("weight gain" OR "weight loss"):ti,ab,kw
 #27 ("lean body mass" or "fat-free mass" or "fat free mass" or "skeletal muscle" or "muscle mass" or sarcopenia):ti,ab,kw
 #28 (malnutrition or malnourished or undernutrition or overnutrition or (nutrition* NEXT assessment*)):ti,ab,kw
 #29 ((antioxidant* or vitamin* or mineral* or micronutrient* or (trace NEXT element*) or electrolyte* or glucose or glutamine or (amino NEXT acid*)) NEAR/2 (deficien* or status)):ti,ab,kw
 #30 (appetite or "energy intake" or "energy expenditure"):ti,ab,kw
 #31 MeSH descriptor: [Basal Metabolism] this term only
 #32 ((bio-electric* OR bioelectric*) NEXT impedance):ti,ab,kw
 #33 MeSH descriptor: [Electric Impedance] this term only
 #34 (#14 OR #15 OR #16 OR #17 OR #18 OR #19 OR #20 OR #21 OR #22 OR #23 OR #24 OR #25 OR #26 OR #27 OR #28 OR #29 OR #30 OR #31 OR #32 OR #33)
 #35 MeSH descriptor: [Nutritional Support] explode all trees
 #36 MeSH descriptor: [Enteral Nutrition] explode all trees
 #37 MeSH descriptor: [Parenteral Nutrition] explode all trees
 #38 MeSH descriptor: [Nutrition Policy] explode all trees
 #39 MeSH descriptor: [Nutrition Therapy] explode all trees
 #40 MeSH descriptor: [Dietetics] explode all trees
 #41 MeSH descriptor: [Diet] explode all trees
 #42 MeSH descriptor: [Gastrostomy] explode all trees
 #43 MeSH descriptor: [Intubation, Gastrointestinal] this term only
 #44 (tube NEXT feed* or nasogastric NEXT tube* or gastrostom*):ti,ab,kw
 #45 ((nasoenteral or enteral or parenteral) NEAR/2 (nutrition or feed*)):ti,ab,kw
 #46 MeSH descriptor: [Food, Fortified] this term only
 #47 MeSH descriptor: [Dietary Supplements] this term only
 #48 MeSH descriptor: [Electrolytes] this term only and with qualifier(s): [administration & dosage - AD, therapeutic use - TU]
 #49 MeSH descriptor: [Glucose] this term only and with qualifier(s): [administration & dosage - AD, therapeutic use - TU]
 #50 MeSH descriptor: [Amino Acids] explode all trees and with qualifier(s): [administration & dosage - AD, therapeutic use - TU]
 #51 MeSH descriptor: [Trace Elements] this term only and with qualifier(s): [administration & dosage - AD, therapeutic use - TU]
 #52 MeSH descriptor: [Micronutrients] explode all trees
 #53 MeSH descriptor: [Dietary Proteins] explode all trees
 #54 MeSH descriptor: [Gastrointestinal Microbiome] this term only
 #55 (nutrition* or nutrient* or diet*):ti,ab,kw
 #56 (oral NEXT (supplement* OR formula?)):ti,ab,kw
 #57 (food* NEXT fortifi*):ti,ab,kw
 #58 ((antioxidant* or mineral* or micronutrient* or (trace NEXT element*) or vitamin* or electrolyte* or glucose or glutamine or (amino NEXT acid*)) NEAR/2 (intake or therap* or supplement*)):ti,ab,kw
 #59 "protein intake":ti,ab,kw
 #60 (microbiome or "gut biome" or "gut microbiota"):ti,ab,kw
 #61 (#35 or #36 or #37 or #38 or #39 or #40 or #41 or #42 or #43 or #44 or #45 or #46 or #47 or #48 or #49 or #50 #52 or #53 or #54 or #55 or #56 or #57 or #58 or #59 or #60)
 #62 (#7 AND #13 AND #34)
 #63 (#7 AND #13 AND #61)
 #64 (#62 OR #63)

Update search run as:

#65 #7 AND #13 AND #61 with Cochrane Library publication date Between May 2024 and Jul 2025

**Ebsco CINAHL**

S1 (MH "Osteosarcoma+")

S2 TX (osteosarcoma* or osteogenic sarcoma*)

S3 (MH "Bone Neoplasms")

S4 TX ("bone tumo#r*" or "bone sarcoma*")

S5 TX ("solid tumo#r*")

S6 TI ((child* or p#ediatric or adolescen* or young) N3 (cancer or oncology))

S7 (S1 or S2 or S3 or S4 or S5 or S6)

S8 (MH "Child")

S9 (MH "Adolescence")

S10 (MH "Pediatrics")

S11 (MH "Young Adult")

S12 TX (child* or children* or p#ediatric* or young adult or adolescen* or teen* or "TYA" or "YA" or girl* or boy* or infan* or juvenil* or youth*)

S13 (S8 or S9 or S10 or S11 or S12)

S14 (MH "Absorptiometry, Photon")

S15 (MH "Pediatric Obesity")

S16 (MH "Body Weights and Measures+")

S17 (MH "Body Composition+")

S18 (MH "Nutritional Status")

S19 (MH "Nutrition Disorders")

S20 (MH "Child Nutrition Disorders+")

S21 (MH "Nutritional Assessment")

S22 (MH "Anthropometry")

S23 TX ("DEXA" or "DXA" or "dual-energy x-ray absorptiometry" or "dual-energy x-ray absorptiometry")

S24 TX ("body composition" or "body weight" or "body size" or "body fat" or "skinfold thickness" or "skin-fold thickness" or "skin fold thickness" or anthropometr* or "mid-upper arm circumference" or "mid upper arm circumference")

S25 TX ("body mass index" or "BMI")

S26 TX ((weight or height) N1 ("z score" or z-score or percentile*))

S27 TX ("weight gain" or "weight loss")

S28 TX ("lean body mass" or "fat-free mass" or "fat free mass" or "skeletal muscle" or "muscle mass" or sarcopenia)

S29 TX (malnutrition or malnourished or undernutrition or overnutrition or "nutrition* assessment*")

S30 TX ((antioxidant* or vitamin* or mineral* or micronutrient* or "trace element*" or electrolyte* or glucose or glutamine or "amino acid*") N2 (deficien* or status))

S31 TX (appetite or "energy intake" or "energy expenditure")

S32 (MH "Basal Metabolism")

S33 TX ("bio-electric* impedance" or "bioelectric* impedance")

S34 (MH "Electric Impedance")

S35 (S14 OR S15 OR S16 OR S17 OR S18 OR S19 OR S20 OR S21 OR S22 OR S23 OR S24 OR S25 OR S26 OR S27 OR S28 OR S29 OR S30 OR S31 OR S32 OR S33 OR S34)

S36 (MH "Nutritional Support+")

S37 (MH "Enteral Nutrition")

S38 (MH "Parenteral Nutrition+")

S39 (MH "Nutrition Policy+")

S40 (MH "Diet Therapy+")

S41 (MH "Dietetics")

S42 (MH "Diet")

S43 (MH "Gastrostomy") OR (MH "Gastrostomy Tubes")

S44 (MH "Intubation, Gastrointestinal")

S45 TX ("tube feed*" or "nasogastric tube*" or gastrostom*)

S46 TX ((nasoenteral or enteral or parenteral) N2 (nutrition or feed*)

S47 (MH "Food, Fortified")

S48 (MH "Dietary Supplementation") OR (MH "Dietary Supplements")

S49 (MH "Electrolytes/AD/TU")

S50 (MH "Glucose/AD/TU")

S51 (MH "Amino Acids+/AD/TU")

S52 (MH "Trace Elements/AD/TU")

S53 (MH "Micronutrients")

S54 (MH "Dietary Proteins")

S55 (MH "Gut Microbiota")

S56 TX (nutrition* or nutrient* or diet*)

S57 TX ("oral supplement*" or formula? or (food* N fortifi*))

S58 TX ((antioxidant* or mineral* or micronutrient* or "trace element*" or vitamin* or electrolyte* or glucose or glutamine or "amino acid*") N2 (intake or therap* or supplement*))

S59 TX ("protein intake")

S60 TX (microbiome or "gut biome" or "gut microbiota")

S61 (S36 OR S37 OR S38 OR S39 OR S40 OR S41 OR S42 OR S43 OR S44 OR S45 OR S46 OR S47 OR S48 OR S49 OR S50 OR S51 OR S52 OR S53 OR S54 OR S55 OR S56 OR S57 OR S58 OR S59 OR S60)

S62 (S7 AND S13 AND S35)

S63 (S7 AND S13 AND S61)

Update search run as:

S7 AND S13 AND S61

Limiters - Publication Date: 20240501-20250731
