# Supplementary material for: Nutrition Support Interventions for Children and Young People Treated for Osteosarcoma: A Scoping Review
Source: J Hum Nutr Diet. 2025 Nov 28;38(6):e70172. doi: 10.1111/jhn.70172 (PMC12661479; doi:10.1111/jhn.70172)
Supplement: Supplementary file 2 — Supplemental Table S2: Data charting form template. [file JHN-38-0-s002.docx]

Supplementary table S2: data charting template

| Variables | Study |
| --- | --- |
| Title |  |
| Citation |  |
| Author |  |
| Year published |  |
| Country |  |
| Publication |  |
| Funding source |  |
| Methodology |  |
| Inclusion criteria/pop studied |  |
| Population and treatment regime for OS |  |
| Sample size |  |
| Age |  |
| Sex |  |
| Primary aim and objectives |  |
| Secondary objectives |  |
| Study design |  |
| Focus of interventions |  |
| Content of interventions/assessment |  |
| Exclusion criteria |  |
| Other demographics/variables |  |
| Primary outcomes and measures (with reference ranges/standards) |  |
| Other outcomes and measures |  |
| Timing and duration of data collection |  |
| Data source |  |
| Dates data collected over/study duration |  |
| Study data collector |  |
| Key findings |  |
| Other outcome findings |  |
| Limitations |  |
| Quality of study design |  |
| Future research priorities identified by authors |  |
