# Supplementary material for: Nutrition Support Interventions for Children and Young People Treated for Osteosarcoma: A Scoping Review
Source: J Hum Nutr Diet. 2025 Nov 28;38(6):e70172. doi: 10.1111/jhn.70172 (PMC12661479; doi:10.1111/jhn.70172)
Supplement: Supplementary file 3 — Supplemental Table S3: Conference abstracts meeting inclusion criteria but excluded at full text characterization. [file JHN-38-0-s001.docx]

Supplementary table S3: Characteristics of conference abstracts meeting inclusion criteria but excluded from analysis against review objectives

| Author, year & country | Study design | OS sample size  Age & sex of cohort | Aims & Objectives | Nutrition Support Intervention Characteristics | Outcomes |
| --- | --- | --- | --- | --- | --- |
| Hazarika M., et al  2022  (India) | Retrospective, single centre | N = 104  OS = 44.2% of total sample  Mean age (years)  13.9 +/- 4.13  M:F: 1.2:1 | Describe the nutritional profile and outcome of OS/Ewings patients with special emphasis on nutritional intervention | High, moderate and low-calorie interventions | World Health Organisation Z-scores and Frisancho chart for mid-upper arm circumference > 5 years |
| Iniesta RR et al 2023  (UK) | Retrospective single centre, service evaluation | N = 11 (100% OS)  Mean age: 13.3 +/2.8 years  Female: 72.7% | Investigate nutrition status and nutrition support during OS treatment and association between NS, EI and protein intake, treatment length and complications | NG, PN, ONS, EN + PN | - Nutrition status at start of treatment: over, well, or undernourished using BMI classification (BMI <-2SD = undernourished, BMI >/= 1 SD overnourished (WHO 2010))  - BMI Z-score change from start to end of treatment  - % cohort requiring ONS, NG, TPN, CNS  - Energy intake, protein intake % of DRV 1991 requirements; |
| O'brien et al 2018  (UK) | Retrospective cohort, single centre observational | N = 33 (100% OS)  0-18 years  Sex NR | Investigate nutritional status and interventions taken in paediatric and adolescent OS undergoing MAP chemo | range of oral, EN, PN interventions | Weight, height, BMI Z-scores at diagnosis and 'through treatment'  Use of ONS, EN, PN incidence of toxicities (nausea, mucositis) through treatment |
| Bajpai et al 2018  (India) | Cohort study Not clear if retrospective or prospective | N = 495 (100% OS)  Median age 17 years  Sex NR | Exploring Non-HD MTX based protocols and aggressive multimodality approach in low- and middle-income setting | ‘Targeted nutritional support if required’ | Tumour necrosis, event-free survival and osteosarcoma toxicities nutrition status (LDH, SAP levels) |
| Novina et al  2022  (Indonesia) | Case study | N = 1 (female)  Age: 5 years | ‘Is Platinum-Based Chemotherapy resulted Hypomagnesemia in a Girl with Osteosarcoma?’ | Mg sulphate IV and oral, calcitriol, vitamin D3 and calcium | ‘Improvement’ post supplementation, otherwise NR |

*BMI: body mass index; EN: enteral nutrition; DRV: dietary reference values; EI: energy intake; F: female; HD MTX: high dose methotrexate; M: male; Mg: magnesium NG: nasogastric; NR: not reported; NS: nutrition support PN: Parenteral nutrition; ONS: oral nutrition support; OS: osteosarcoma
