# Supplementary material for: Nutrition Support Interventions for Children and Young People Treated for Osteosarcoma: A Scoping Review
Source: J Hum Nutr Diet. 2025 Nov 28;38(6):e70172. doi: 10.1111/jhn.70172 (PMC12661479; doi:10.1111/jhn.70172)
Supplement: Supplementary file 4 — Supplemental Table S4: Full table of key findings, limitations and critical appraisal of included full text sources. [file JHN-38-0-s004.docx]

**Table S4a: Characteristics of full text sources of evidence reporting on comprehensive nutrition support for CYP undergoing treatment for OS (total cohort data, stratified for OS only where specified)**

| **Author, year and country** | **Study design** | **Sample size and sex**  **(n =total cohort**  **OS = OS cohort)** | **Age at diagnosis (years)** | **Diagnosis & OS treatment protocol** | **Study aims & objectives** | **Nutrition intervention content, route, timing, dose, regimen** | **Outcomes and data source** | **Outcome reporting methods** |
| --- | --- | --- | --- | --- | --- | --- | --- | --- |
| Henry et al. 2017  (France) | Retrospective multi-centre cohort | n = 138  OS = 59  OS sex NR  OS GT n = 31  OS NG n = 4  OS no GT/NG n = 24 | Mean ±SD  eGT: 13 ± 3.8  NG: 11.4 ± 4  No eGT/NG: 11.9 ± 3.5  OS age NR | OS  EW  Protocol  OS94  OS2006 | Comparison of EN via eGT  vs NG vs no eGT/NG | 3 NI groups:  - eGT  - NG  - No eGT/NG | - Primary: Nutritional status  Complications  - Secondary:  Treatment modifications  EFS at 4 years  Data from medical records | - BMI and Waterlow Z score changes over treatment  - Frequency of hospitalisations  - Frequency of early GT complications: minor and major (method: clinical judgement and treatment required for complication) |
| Schmitt et al. 2012 (France) | Retrospective multi-centre cohort | N = 48  OS = 31  Gp 1 Males = 15  Gp 2 Males = 10  *p* = NR  OS sex NR | Median (Range)  Gp 1: 13.3 (8.2-17.3)  Gp 2: 14.3 (9.3-18.9)  OS age NR | OS  EW  Protocol OS 2006 | Efficacy of early EN protocol (PEG insertion at diagnosis) vs. no systematic EN protocol | Gp 1: eGT  - EN given overnight +/- daytime bolus  - Ad libitum oral diet  - EN volume adapted to clinical tolerance  Gp 2: no early EN protocol | Primary: Nutrition status  Secondary: Clinical status  Cancer progression  Data source NR | - Waterlow(1) scores: wt for ht and ht for age Z scores.  - Incidence of complications after tumour resection: local site complications, delayed wound-healing, tumour operative site infection  - Relapse rate  -Cancer-related deaths |
| Richioud, et al 2015 (France) | Single centre retrospective cohort | n = 11  OS = 6  Male n = 7  OS Male n = 3 | Median (Range): 13 (3-20) years  OS: 16 (10.5-20) years | OS  UCN  PA  MNBL  Protocol NR | Safety and effectiveness of primary placement button RIG in children with cancer | Enteral feeding via GT introduced 18h after insertion.  Feed product/volume adapted to needs of child, ↑over 5 days up to 100% dose during cyclized injections (Mean 10 h/day) | Nutrition status Safety of primary RIG button placement  Data from patient files | - Wt/ht ratio percentage: actual BMI as percentage of ideal BMI (WHO child growth standards)(2).  - Incidence of major complications: peritonitis, haemorrhage, gastro-cutaneous fistula, wound infection, necrotizing fasciitis, inadvertent removal - Incidence of minor complications: leakage, blockage, granulation tissue formation, pain |
| Dalton & Johnson 2024 (USA) | Single centre retrospective cohort | N = 58  OS = 17  Sex NR | Age NR | NBL, OS, EW, RB, Wilms, HBL | Document incidence and risk factors for GT site erythema concerning for infection | NR | Cancer diagnosis  %wt loss at diagnosis/ relapse  GTs placed at diagnosis vs after therapy starts  GT type/placement location  Incidence of erythema  Incidence of:  - neutropenic erythema  - therapy delays due to erythema  Use & type of antimicrobial prophylaxis  Data from patient records | Frequency of Corpak or Mic-Key button placement  Frequency of placement in theatre or radiology  -Erythema incidence via qualitative assessment of records from experienced nurse practitioners. No standard documentation available  -% patients with erythema given antibiotics |
| Ringwald-Smith et al 2011 (USA) | Single centre, retrospective cohort | n = 48  OS = 48  Male n = 25 | Median 13.7 (3.2 -23.00) | OS  ProtocolOS99 | Identify NI types provided during treatment and effectiveness at improving weight status | Appetite stimulants (dronabinol, cyproheptadine,  megestrol),  Enteral nutrition, Parenteral nutrition | Primary: Types of NI during treatment  Effectiveness of NIs to maintain/improve weight status  Data from clinical charts | -Frequency of type of NI provided  -Maintenance of, or movement between, BMI Z-scores translated into weight categories: Underweight; Healthy weight; Overweight; Obese (https://www.cdc.gov/bmi/child-teen-calculator/) |
| Levin, 2017  (USA) | Case report | N = 1 | 10-year-old male | OS  ASD  Protocol AOST0331 | Summarise nutritional experiences of autistic child through treatment | GT </= 1 month of diagnosis; 1 Kcal/ml peptide feed. Fed throughout treatment and beyond. Nocturnal + daytime pumped boluses (50-100% TER), ad libitum oral diet | Outcomes: nutrition status changes over treatment  Nutrition-related clinical signs and symptoms  Data source NR | Measures: BMI Z-score evolution over treatment. |

ASD: autistic spectrum disorder; BMI: Body Mass Index; CYP: Children and young people; eGT: early gastrostomy; EN: Enteral nutrition; EFS: Event-free survival; EN: enteral nutrition; EW: Ewing sarcoma; Gp: Group; GT: gastrostomy; ht: height; MNBL: metastatic neuroblastoma; NG: nasogastric; NI: Nutrition interventions; NR: Not reported; NS: nutrition support; OS: Osteosarcoma; PA: Pilocytic astrocytoma; RIG: radiologically inserted gastrostomy; TER: total energy requirements based on age, sex and weight; UCN: Undifferentiated carcinoma of nasopharynx; VS: versus; Wt: weight

**Table S4b Key findings and limitations of individual sources of evidence (Comprehensive nutrition support)**

| **Authors** | **Study design and Quality** | **Measured outcomes** | **Results (for full cohort, stratified for OS only where specified)** | | | **Critical appraisal** |
| --- | --- | --- | --- | --- | --- | --- |
|  |  |  | **At diagnosis** | **During treatment** | **End of treatment** |  |
| Henry et al  2017 | Retrospective multi-centre cohort  Weak | BMI and Waterlow Z scores as a function of time during treatment  Frequency/type GT complications  Hospitalisation frequency and durations for GT or other non-planned complications  Pre and post-op CT modification  Time between pre-op CT and surgery and surgery and post-op CT  Total treatment duration  EFS at 4 years  Frequency and site of relapse  Days on PN | **Primary outcomes:**  BMI Z-score 0.1 ± 1.4 SD  eGT -0.30 (SD NR)  NG -0.52 (SD NR)  No eGT/NG 0.25 (SD NR)  GT complication frequency (%):eGT: n = 37 (63.8)  GT Hospitalisations  eGT: n = 4 (OS)  Major n = 1  Severe n = 3  OS GT vs EW NG *p* = 0.02  OS eGT: 31 (53.4%)  EW NG: 16 (80%) | **Primary outcomes**  BMI Z score changes (diagnosis – 6/12 months) (*p* = 0.09) Surgery: eGT -0.32 (SD NR); NG -0.70 (SD NR); No eGT/NG -0.34 (SD NR)  3 months after surgery: eGT -0.40 (SD NR); NG -0.65 (SD NR); No eGT/NG -0.30 (SD NR)  **Secondary outcomes:**  Hospitalisation for CPs (mean ±SD)  Total hospitalisations per patient (*p* = 0.18)  eGT 5.3 ± 2.8; NG 6.3 ± 3.4; No eGT/NG 4.7 ± 2.6  Hospitalisation duration (days) (*p* = 0.15)  eGT 29.9 ± 24.4; NG 38.0 ± 20.3; No eGT/NG 29.9 ± 22.5  Hospitalisations for:  Febrile aplasia (*p* = 0.34): eGT 4.1 ± 2.4; NG 5.3 ± 3.3; No eGT/NG 4.1 ± 2.6  Surgery CPs (*p* = 0.28): eGT 0.5 ± 0.8; NG 0.2 ± 0.5; No eGT/NG 0.3 ± 0.6  Mucocutaneous CPs (*p* = 0.19): eGT 1.0 ± 1.2; NG 1.6 ± 1.6; No eGT/NG 1.0 ± 1.1  Time between treatment stages in months (Mean ± SD)  Pre-op CT to surgery (*p* = 0.06)  eGT 3.9 ± 0.8; NG 4.5 ± 0.9; No eGT/NG 4.0 ± 0.7  Surgery to post-op CT  eGT 0.5 ± 0.5; NG 0.5 ± 0.2; No eGT/NG 0.6 ± 0.3  Pre-op CT regimen changes:  CT delays (p = 0.67): eGT n = 12; NG n = 6; No eGT/NG n = 14  CT changes (p = 0.11): eGT n=23; NG n=3; No eGT/NG n=18  Post-op CT regimen changes:  CT delays (p = 0.06): eGT n=39; NG n=9; No eGT/NG n=29  CT changes (p = 0.04): eGT n=34; NG n=8; No eGT/NG n=22 | **Primary outcomes**  BMI Z score 6 – 12 months after treatment:  eGT 0.10 (SD NR)  NG (SD NR)  No eGT/NG -0.34 (SD NR)  *p* = 0.23 (time x group)  *p* = 0.09 (time x group – adjusted by diagnosis BMI Z)  No differences in EFS at 4 years (*p* = 0.64)  Probability of survival (*p = 0.31)*  eGT n = 43 (74%)  NG n = 17 (85%)  No eGT/NG n = 41 (68%)  ‘Average’ days on PN  (*p* = 0.0038):  eGT: 5.8 ± 14.2  NG: 9.9 ± 10.4  No eGT/NG: 8.5 ±13.9 | - Did not record NG-related complications as a confounder  - Non normally distributed variables should be reported as median (IQR)  - No information on the following:  Age at 1^st^ measurement  Statistical analysis for BMI Z score (group x time) NR  - All outcomes to be measured were reported but precise data reporting incomplete  - Study design for feasibility (no validated guideline)  - No validated checklist used such as STROBE or RECORD  - Statistical analysis chosen should account for cofounding factors e.g. different OS treatment protocols, differences in diagnoses of each NS group, resection margins and days on PN, covariates or mediators and the differences in sample size between groups and time points  - Use of validated tool for assessing complications NR |
| Schmitt et al  2012 | Retrospective multi-centre cohort analysis  Weak | Age/sex at presentation  Symptoms at presentation NR  OS: Ewings ratio  Type of treatment  Delay, duration and complications of PEG  Evolution of W/H and W/A Z scores during treatment and follow up  Long term evolution of disease | Age (years) Median (range) NS *p* NR  Gp 1: 13.3 (8.2-17.3)  Gp 2: 14.3 (9.3-18.9)  Sex ratio (%): NS (*p* NR)  Gp 1: Male = 15 (52%)  Gp 2: Male = 10 (53%)  OS/Ewing ratio: NS (*p* NR)  Gp 1: 18/11  Gp 2: 13/6  W/H and W/A Z score NS (*p* NR)  Gp 1: -0.3 (-2.1 - 2.3)  Gp 2: -0.8 (-2.6 - 9.4)  H/A Z score: NS (*p* NR)  Gp 1: 0.8 (-2.0 - 2.1)  Gp 2: 0.2 (-1.3 - 2.4)    % Metastasis NS (*p* NR)  Gp 1: 24.1; Gp 2: 36.8 | % weight change at 6 months Mean ± SD): NS *p* NR  Gp 1: -0.1% ± 7.1%; Gp 2: -4.7% ± 8.7%  Median W/H Z-score at 6 months:  Gp 1 reported no ↓ (*p* NR)  Gp 2: -1.4 SD reported NS (*p* NR)  Median time before surgery: NR *p* NR  Gp 1: 118 (73-159)  Gp 2: 123 (102-225)  Bad response in histopathology (% Rosen I/II) NS *p* NR  Gp 1: 18.5; Gp 2: 16.7  Efficacy of surgery (% R0) NS *p* NR  Gp 1: 88.9; Gp 2: 89.5  Post tumour resection complications NR *p* NR:  Gp 1: n = 3; Gp 2: n = 4  Gp 1 local PEG complications: n = 27  | Final W/H Z scores NS (NR)  H/A Z score NS NR  Chemotherapy duration Median (range) months NS *p* NR  Gp 1: 10 (4.3-16.3)  Gp 2:11.9 (4.1-14.2)  Median (range) follow up months: NS *p* NR  Gp 1: 22 (5-63)  Gp 2: 26 (5-53)  % Relapse rate: NS (*p* = 0.69)  Gp 1: 31; Gp 2: 47  % Cancer-related deaths NS (*p* = 0.07)  Gp1: 3; Gp 2: 26 | - Statistical analysis chosen should account for confounding factors, covariates or mediators and the differences in sample size between groups and time points  - Incomplete statistical data e.g. missing *p* values to report statistical significance  - Validated tool for assessment of complications needed e.g. PEG  - No reporting validated checklist used e.g. STROBE  - Not all outcomes reported e.g. symptoms at presentation |
| Richioud et al 2015 | Service evaluation  (single centre retrospective cohort)  Moderate | Cancer diagnosis  Age, wt, ht at diagnosis, at GT insertion, 1, 2 and 3 months  Delay between diagnosis and GT procedure  Number and type of CT before and after GT  Procedural and post-procedural complications | Diagnoses: OS n = 6,  UCN n = 3; PA n = 1  MNBL n = 1  Median (range):  Age in years: 13 (3-20)  OS*: 16 (10.5-20)  Wt in kg: 40 (10.4-69.2)  OS*: 54.25 (26.6-69.2)  Ht* in cm: 146 (89-180)  OS*: 166cm (141-180)  % W/H ratio*:  98.7 (78.5-121.8)  OS*: 96.1 (78.5-121.8) | Time between diagnosis and GT: median est 2 months (1-9)  Number of CT cycles before GT (OS)*: 5.5 (2-11)  Type of CT cycles at GT insertion (OS):  Methotrexate/ifosfamide/ etoposide Adriblastin/cisplatin/holoxan  Number and type of CT cycles after GT: NR  Major procedural and post-procedural complications: n = 0  Minor complications: n =2  1 month post GT: Wt gain: n = 7; Wt loss: n = 1  Maintained weight: n = 2  3 months post GT: Wt gain n = 8; Wt loss n = 1 | N/A | - W/H ratio changes between diagnosis and GT and at 2 months reported in graphic form only  - *Data summarised from tables by review authors not study authors  - Classified complications using Clinical Practice Guideline: General principles for evaluation of new interventional techniques, Society of Interventional Radiology.  - Unclear if complete and consecutive inclusion of cases  - Reports of clinical site and participants demographic data limited  - Clinical information and outcomes of participants clearly reported  - Clear criteria for inclusion  - Procedures and outcomes described in a reliable and measurable way, but not all outcomes reported |
| Dalton & Johnson2024 | Single centre retrospective cohort  Weak | Cancer diagnosis  %wt loss at diagnosis/relapse  GT type, timing and placement location  Incidence of erythema concerning for infection  Incidence of:  - neutropenic erythema  - therapy delays due to erythema  Use & type of antimicrobial prophylaxis | Diagnoses: NBL n = 24, OS n = 17; EW n = 9, RB n = 6, Wilms n = 1, HBL n = 1  % weight loss NR  GT placement at diagnosis n = 25 (43%)  OS n = 16 (95% of OS) | GT placement after therapy started n = 33 (57%)  GT placement location: interventional radiology n = 7 (12%)  Operating room: n = 51 (88%)  GT type: Corpak n = 3 (5%)  Mic-Key button n = 55 (95%)  Erythema concerning for infection: n = 31 (53%)  OS erythema concerning for infection: n = 5 (29% of 17)  Of total erythema cases:  - Erythema for GTs placed after therapy started: n = 23* (74%)  - Erythema during neutropenia: n = 22* (71%)  - Mic-Key buttons placed in operating room NR  Cancer therapy delay due to erythema: n = 1 (3%)  Erythema cases not given prophylactic antibiotics after 1^st^ episode: n = 27 (87%)  Prophylactic clindamycin used n = NR | N/A | Demographic reports limited e.g. age, sex, co-morbidities NR. Clear description of clinical site.  Clear criteria for inclusion/exclusion  Qualitative assessment of erythema by authors – no validated tool  Not all outcomes reported  *Some data summarised by review authors as incompletely reported |
| Ring-wald Smith et al 2011 | Service evaluation (single centre retrospective cohort)  Moderate | 4 data points: diagnosis, week 12, 23, after EOT  Age/sex with NI  Ht and wt at each data point  NI type/duration in 30 days before data point  Effectiveness of NI (transition between  BMI categories) | Median age (years) (range) and NI (p<0.001)  With NI = 9.9 (6.5-15.0)  No NI: 15.5 (3.2-23.0)  Sex and NI (*p* = 0.21)  With NI (M;F): 5;9  No NI: (M;F) 20;14  Any NI provided at any time points: n = 14  No. patients given NI (at diagnosis):  AS: n = 1PN: n =1  EN: n =1  AS + PN: n = 0 | Number of patients given NI week 12:  AS n = 3  PN: n = 1  EN: n = 0  AS + PN: n = 2  No. patients given NI week 23:  AS: n = 8  PN: n = 0  EN: n = 0  AS + PN: n = 4  NI and BMI category *(p= 0.005)*  NI given when underweight: n= 4  NI not given when underweight: n=0  Ht and wt at each time point (NR) | After EOT:  AS n = 3  PN n = 0  EN: n = 0  AS + PN: n = 0  Duration of any NI across all time points (days) (Median) (range): 30 (1-30)  No. patients NI was effective for: 7**  Ineffective: 7  (*p* NR) | - Definition and content of each nutrition support interventions NR  - Exclusion reasons described i.e. insufficient information for complete evaluation  - Effectiveness of nutrition intervention data reported differently in data summary compared to tabular data  - Using validated outcomes measures for effectiveness of nutrition interventions?  - ** inconsistent/inaccurate data reporting? Reported as 11 and 3 in paper |
| Levin, 2017 | Case report  Strong | Planned outcomes NR | BMI Z scores for age and sex: -1.63,  Early GT for EN, continued until 1 year post treatment | BMI Z-score: - 0.41 SD pre-surgery | Osteoporosis and vitamin D deficiency: given 50,000IU/wk x 8    BMI -0.54 at EOT (corrected for limb amputation) | - Demographics (incomplete), history,  diagnostic tests, assessments clearly described  - Extensive description of nutrition status throughout treatment, content of NI and post-NI clinical/nutritional status. Adverse events described.  - Takeaway lessons provided |

AS: appetite stimulant; BMI: body mass index; CP: complications; CT: Chemotherapy; eGT: early gastrostomy; EFS: event-free survival; EN: Enteral nutrition EOT: End of treatment; EW: Ewing sarcoma; Gp: Group; GT: Gastrostomy; H/A: height for age; HBL: hepatoblastoma; NBL: neuroblastoma; NI: Nutrition intervention; NR: Not reported; NS: Not significant; PA: Pilocytic astrocytoma; PN: parenteral nutrition; RB: rhabdomyosarcoma; UCN: undifferentiated carcinoma of nasopharynx; W/A weight for age W/H weight for height; Wk: week; Wt: weight

**Table S4c Characteristics of full text sources of evidence reporting on micronutrient support for CYP undergoing OS treatment (total cohort data, stratified for OS only where specified)**

| **Authors and country** | **Study design** | **Total Sample size (n), OS cohort and sex** | **Age at diagnosis (years)** | **Diagnoses & OS treatment protocol** | **Study aims** | **Nutrition intervention content, route, dose** | **Outcomes** |
| --- | --- | --- | --- | --- | --- | --- | --- |
| Lee et al 2023  (China) | Case series | n =3  OS n = 2 (1M, 1F) | 12; 17 | OS; HUS  Protocol NR | Raise awareness of paediatric (WE) by describing local cases | TPN for 1 week and 2 months    1000mg and 1500mg Th given daily (route and duration NR) to each case | - Symptoms at presentation  - TPN duration prior to onset  - MRI findings  - Laboratory findings post Th replacement  - Clinical outcome |
| Perko et al 2012  (USA) | Case series | n = 5  OS n = 1 (F) | 9 | MBL; OS; AML; PR  Protocol NR | Highlight risk of developing WE during IV MVI shortages Importance of early diagnosis | TPN  IV Th (dose, route and duration NR) | - Clinical status by MRI brain 7 days post Th replacement |
| Castelan-Martinez et al 2020  (Mexico) | RCT | n = 46  OS = 63/101 CT cycles  F = 62 cycles | Median (IQR):  13.5 (3.6) | OS; GCT; HBL; EP  Protocol NR.  CT given: CIS, DOX, MTX; CP, ET, IF, VC, 5-F | Assess efficacy and safety of oral Mg to reduce FN episodes, hypoMg and septic shock in paediatric patients with solid tumours treated with cisplatin | Oral Mg 250mg daily from start of admission (end date NR) | - Incidence of FN  - Incidence of hypoMg  - Incidence of septic shock |
| Nozaki et al 2001  (Japan) | RCT | n = 29 (all OS)  Intervention Gp:  M = 15; F = 3  Control: M = 5; F = 6 | Median (range):  19 (9-58) | OS protocol NR. Included surgery + pre and post DOX + CIS | Examine survival prognosis of patients with OS given oral aD3 compared to those without | Oral aD3 (0.75 - 4 µg) for mean 25 months (SD NR)  Oral aD3 > 1500µg total dose  Oral aD3 4µg daily > 6months | Percentage 5- and 10-year survival rates and survival time (in months) |

aD3: active Vitamin D3; AML: Acute myelogenous leukaemia; CIS: Cisplatin; CT: Chemotherapy; CP: Cyclophosphamide; CYP: children and young people; DOX: Doxorubicin/Adriamycin; ET: Etoposide; EP: Ependymoma; F: Female; FN: Febrile neutropenia; Gp: group; GCT: Germ cell tumour; HBL: Hepatoblastoma; HUS: Haemolytic uraemic syndrome; HypoMg: hypomagnesemia; IF: Ifosfamide; MBL: medulloblastoma; Mg: magnesium; M: Male; MTX: Methotrexate; NR = Not reported; OS = Osteosarcoma; PR: Parameningeal rhabdomyosarcoma; RCT: randomised controlled trial; SD = Standard deviation; Th: thiamine; TPN = Total parenteral nutrition; VC: Vincristine; WE = Wernicke’s encephalopathy; 5-F: 5-Fluorouracil

**Table S4d** **Key findings and limitations of individual sources of evidence for micronutrient support**

| **Study** | **Study design & Quality** | **Measured outcomes** | **Results** | **Critical appraisal** |
| --- | --- | --- | --- | --- |
| Lee et al 2023 | Case series  Moderate | Symptoms at presentation (OS)  TPN duration prior to onset (OS)  MRI findings (OS)  Laboratory findings (post Th supp) (OS)  Clinical outcome (OS) | Presentation: Confusion, athetoid movements n =1; Dullness, flaccid tones n = 1  TPN duration prior to onset: 1 week n = 1; 2 months n = 1  MRI findings: Dorsomedial thalamic T2 hyperintensity and restricted diffusion n = 1  T2 hyperintensity and restricted diffusion at mammillary bodies, thalami and periaqueductal grey matter; cerebral cortex involvement; contrast enhancement in mammillary bodies n = 1    Lab findings post Th supp: Normal/↑ Transketolase: n = 2; Normal Th diphosphate n = 2  Clinical outcome: Complete resolution of symptoms n = 1; Improved mental status but limited motor control n = 1 | - Condition measured reliably and consistently for all subjects  - Valid method used for identification of condition  - Clear reporting of clinical information of participants and outcome  - Description of nutrition intervention (TPN and thiamine) incomplete  - Unclear if complete or consecutive inclusion of all cases; minimal demographic reporting of presenting site and study population |
| Perko et al 2012 | Case series  Weak | Underlying diagnosis  Clinical presentation  Lab findings  MRI findings before and after Th replacement | Diagnosis: OS with IV MTX 9 days before presentation and TPN during IV multivitamin shortage. Presentation: Somnolence with incoherent speech  Lab findings at presentation: Lactate: 8.9mmol/L (high); Th: 43 nmol/L (low)  MRI before Th supp: T2 prolongation of medial thalami, mammillary bodies, dorsal pons  After 7 days Th: decreased T2 prolongation involving subthalamic nuclei and mammillary bodies but increased T2 prolongation involving bilateral caudate heads  Clinical status after Th supp: Remained hospitalized for 80 days. Neurological status gradually returned to baseline | - Valid method used for identification of condition  - Incomplete lab results reporting after Th supp  - Incomplete reporting of TPN intervention  - Incomplete reporting of Th intervention  - Unclear if complete or consecutive inclusion of all cases and incomplete demographic reporting of clinical site and patient |
| Castelan-Martinez et al 2020 | RCT  Strong | Incidence of:  - FN  - hypoMg  - septic shock  Serum Mg in Mg group with/without FN  CTCAE level of hypoMg  Serum K and Ca end of F/U | FN incidence (n) *(p* = 0.019): Mg group = 14; Control = 27; RR (95% CI) = 0.53 (0.32-0.89)  HypoMg incidence (n): Mg group = 7; Control = 10; RR = 0.71 (0.3-1.73)  Septic shock incidence (n): Mg group = 5; Control = 14; RR = 0.43 (0.02-0.94)  Time to FN (days) *(p* = 0.031): Mg group = 25.2; Control = 20.8  Median serum Mg in Mg supplemented group (mg/dL) *(p* = 0.008):  Patients with FN = 1.7; Patients without FN = 1.9; Control group NS *p* NR  HypoMg episodes (n) CTCAE Level 1 = 17; Other levels = 0  Median K level at end of follow up (mEq/L) *(p*= 0.87): Mg group = 3.70; Control = 3.70  Median Ca level at end of follow up (mg/dL) *(p* = 0.61): Mg group = 9.0; Control = 8/8 | - Reasons for exclusion clearly reported  - True randomisation  - Baseline clinical (including nutritional) demographics reported. Socio-demographics NR  - Results adjusted for baseline differences e.g. 5-fluoruracil  - Individual follow up of AE and study compliance; AE classified using validated tool (CTCAE)  - Biases related to unblinded methodology, small sample size, one country/centre, few tumours, no placebo |
| Nozaki et al 2001 | RCT  Weak | 5- and 10-year survival rate with vs without oral aD3  Survival time  Survival rate with total aD3 dose > 1500µg  Survival rate with aD3 4µg daily >6 months | 5-year survival (%) (*p* NR): aD3 group = 61.1; Control = 63.6  10-year survival (%) NS (*p* = 0.3823): aD3 group = 61.1; Control = 33.9  Median (range) survival time (months) (*p* = NR): aD3 group = 88 (17-155)  Control = 38 (6-180)  % Survival with total dose >1500µg: aD3 group: 5-years = 80.0; 10-years = 80.0  Median (range) survival time (months) NS (*p* = 0.0740): aD3 group = 117 (21-138); Control = NR  % Survival with aD3 4µg daily > 6 months: aD3 group: 5-yrs = 75.0; 10-years = 75.0  Median survival time (months) NS (*p* = 0.3387): aD3 group = 98 (21-138); Control = NR | - Small samples size, unblinded methodology, adverse effects not reported, no placebo  - Incomplete baseline clinical, nutritional and socio-demographic reporting  - Randomisation method NR  - Reports baseline differences between groups not significant but *p* NR  - Incomplete statistical analysis reports |

aD3: Active vitamin D3; AE: adverse events; Ca: Calcium; CTCAE: National Cancer institute Common Terminology Criteria for Adverse Events; FN: febrile neutropenia; HypoMg: hypomagnesemia; K: potassium; Lab: laboratory; MRI: magnetic resonance imaging; MTX: Methotrexate; supp: supplementation; Th: Thiamine; TPN: Total parenteral nutrition; %: percent

1. Waterlow JC. Note on the assessment and classification of protein-energy malnutrition in children. Lancet. 1973;2(7820):87-9.

2. WHO Child Growth Standards. Interpreting Growth Indicators. Geneva: World Health Organisation; 2008.
